# Neutrophil-dendritic cell interaction plays an important role in live attenuated *Leishmania* vaccine induced immunity

**DOI:** 10.1371/journal.pntd.0010224

**Published:** 2022-02-22

**Authors:** Parna Bhattacharya, Nevien Ismail, Ankit Saxena, Sreenivas Gannavaram, Ranadhir Dey, Timur Oljuskin, Adovi Akue, Kazuyo Takeda, James Yu, Subir Karmakar, Pradeep K. Dagur, John Philip McCoy, Hira L. Nakhasi

**Affiliations:** 1 Division of Emerging and Transfusion Transmitted Disease, Center for Biologics Evaluation and Research Food and Drug Administration, Silver Spring, Maryland, United States of America; 2 Flow Cytometry Core, National Heart, Lung, and Blood Institute, National Institutes of Health, Bethesda, Maryland, United States of America; 3 Division of Bacterial, Parasitic, and Allergenic Products, Center for Biologics Evaluation and Research Food and Drug Administration, Silver Spring, Maryland, United States of America; 4 Division of Blood Components and Devices, Center for Biologics Evaluation and Research Food and Drug Administration, Silver Spring, Maryland, United States of America; University of Notre Dame, UNITED STATES

## Abstract

**Background:**

Neutrophils are involved in the initial host responses to pathogens. Neutrophils can activate T cell responses either independently or through indirect involvement of Dendritic cells (DCs). Recently we have demonstrated direct neutrophil-T cell interactions that initiate adaptive immune responses following immunization with live attenuated *Leishmania donovani* centrin deleted parasite vaccine (*LdCen*^-/-^). However, neutrophil-DC interactions in T cell priming in vaccine immunity in general are not known. In this study we evaluated the interaction between neutrophils and DCs during *LdCen^-/-^* infection and compared with wild type parasite (*LdWT)* both *in vitro* and *in vivo*.

**Methodology/findings:**

*LdCen^-/-^* parasite induced increased expression of CCL3 in neutrophils caused higher recruitment of DCs capable of inducing a strong proinflammatory response and elevated co-stimulatory molecule expression compared to *LdWT* infection. To further illustrate neutrophil-DCs interactions *in vivo*, we infected LYS-eGFP mice with red fluorescent *LdWT/LdCen^-/-^* parasites and sort selected DCs that engulfed the neutrophil containing parasites or DCs that acquired the parasites directly in the ear draining lymph nodes (dLN) 5d post infection. The DCs predominantly acquired the parasites by phagocytosing infected neutrophils. Specifically, DCs containing *LdCen^-/-^* parasitized neutrophils exhibited a proinflammatory phenotype, increased expression of costimulatory molecules and initiated higher CD4^+^T cell priming *ex-vivo*. Notably, potent DC activation occurred when *LdCen*^-/-^ parasites were acquired indirectly via engulfment of parasitized neutrophils compared to direct engulfment of *LdCen*^-/-^ parasites by DCs. Neutrophil depletion in *LdCen^-/-^* infected mice significantly abrogated expression of CCL3 resulting in decreased DC recruitment in ear dLN. This event led to poor CD4^+^Th1 cell priming *ex vivo* that correlated with attenuated Tbet expression in ear dLN derived CD4^+^ T cells *in vivo*.

**Conclusions:**

Collectively, *LdCen^-/-^* containing neutrophils phagocytized by DC markedly influence the phenotype and antigen presenting capacity of DCs early on and thus play an immune-regulatory role in shaping vaccine induced host protective response.

## Author summary

Visceral Leishmaniasis (VL), caused by the protozoan parasites of the genus *Leishmania* is a neglected tropical disease. *Leishmania donovani* is the principal causative agent of VL in East Africa and the Indian subcontinent whereas in Europe, North Africa, and Latin America VL is mainly caused by *Leishmania infantum*. No licensed vaccine exists against VL. We have reported previously that live attenuated centrin gene-deleted *L*. *donovani* (*LdCen^-/-^*) parasite vaccine induced strong innate immunity which leads to a protective Th1 response in animal models. We recently demonstrated that neutrophils play an indispensable role following immunization with *LdCen*^-/-^ parasites in inducing protective Th1 immune response. However, neutrophils also secrete chemokines that attract other innate cells such as dendritic cells and regulate their activities. In the current study we analyzed the interplay between neutrophils and DCs, and its effects on T cell activation during *LdCen^-/-^* infection and compared with wild type parasite (*LdWT)* infection. We observed that higher recruitment of DCs occurred in *LdCen^-/-^* infected mice ear draining lymph nodes compared to *LdWT*. This recruitment is facilitated by increased secretion of the chemokine CCL3 by neutrophils. A markedly decreased DC recruitment was observed in *LdCen^-/-^* infected mice following CCL3 neutralization indicating the key role of neutrophils in DC recruitment. Further, we demonstrated that DCs that ingest *LdCen^-/-^* infected neutrophils are better activated than those that acquire the parasites independent of neutrophils. Notably neutrophil depletion in *LdCen^-/-^* infected mice also attenuated activation of DCs in the ear dLN that resulted in poor CD4^+^T cell priming. Our results reveal that interaction between neutrophils and DCs play an important role in shaping proinflammatory immune response induced by a live attenuated *Leishmania* vaccine.

## Introduction

Leishmaniasis, caused by the infection with the *Leishmania* protozoa, is endemic to the tropical and subtropical regions of the world. Over 12 million people are currently infected with *Leishmania* with an annual incidence of approximately 2 million new cases and ~50, 000 deaths in approximately 100 countries throughout the world [1,2].

Transmitted by the bite of an infected sand fly, *Leishmania* parasites establish chronic infections by subversion and attenuation of the microbicidal functions of the host phagocytic cells [3]. Neutrophils are the first innate cells recruited to the dermal site of *Leishmania* infection where they play a preeminent role in the phagocytosis and elimination of the parasites via the generation of oxygen intermediates and the release of lytic enzymes stored in their granules [4]. Neutrophils markedly control the development of the adaptive immune response either by direct activation of T cells [5,6] or indirectly via modulation of dendritic cell mediated T cell response in various infections including Leishmaniasis [7–10]. The crosstalk between neutrophils and dendritic cells at the site of infection following the influx of neutrophils to the inoculation site within the first day of infection has been reported to shape the development of the *Leishmania* specific immune response [9,11,12].

Previously, we reported that live attenuated centrin gene deleted *Leishmania donovani* parasites (*LdCen^-/-^*) induced protection against virulent *L*. *donovani* infection in mice, hamsters, and dogs as indicated the induction of a host protective T cell response followed by control of parasite burden [13–16]. Recently, we have demonstrated that *LdCen^-/-^* parasites induced a strong effector function in neutrophils via induction of Neutrophil Extracellular traps (NETs) and production of proinflammatory cytokines. Additionally, neutrophils as antigen presenting cells play an essential role in inducing heightened CD4^+^Th1 response that mediates protection against virulent challenge in *LdCen*^−/−^ immunized mice [6]. Recently, we reported that neutrophils can efficiently influence T cell differentiation [6]. A predominant immunoregulatory role for neutrophils in activating DC functions and enabling T cell differentiation via DCs during *Leishmania* infection has also been reported. For example, in *L*. *major* infection, neutrophils have been found to be critical in the recruitment of DCs and initiation of protective immune response in the resistant C57Bl6 mice [8]. In contrast, other studies have demonstrated that capture of infected neutrophils by dendritic cells during *L*. *major* infection inhibits DCs early anti-leishmania response both *in vitro* and *in vivo* [9,11,12]. Importantly, the low efficacy of killed *Leishmania* vaccines against sand fly challenge was hypothesized to be directly related to the suppressor effects of neutrophils on dermal DCs [17]. However, little is known about the regulatory role for neutrophils in DC function in mediating *Leishmania* vaccine induced immunity.

In the current study we demonstrated neutrophil mediated immunomodulation of DC function and subsequent T cell priming in response to live attenuated *LdCen*^−/−^ vaccine. We showed that following intradermal (ID) immunization with *LdCen*^−/−^, neutrophils release elevated levels of the chemokine CCL3 with potent chemotactic activity for DCs. Such recruited DCs were proficient in inducing a strong proinflammatory response and co-stimulatory molecule expression in ear draining lymph node (dLN) 5d post infection. Further, we demonstrated that DCs that ingest *LdCen^-/-^* infected neutrophils are better activated than those that acquire the parasites independent of neutrophils. Importantly, neutrophil depletion in *LdCen^-/-^* infected mice significantly abrogated the recruitment and activation of DCs in the ear dLNs, and such DCs exhibited poor CD4^+^T cell priming *ex-vivo*. Even though both *LdWT* and *LdCen*^-/-^ parasites were preferentially acquired by a neutrophil-dependent mechanism by the DCs, clear differences in their activation were observed in the two infections. Our study thus reveals a previously unrecognized regulatory role for neutrophils in DC function during *L*. *donovani* infection and suggest that cross-talk between these innate cell populations is an important component of the protective immune response induced by a live attenuated *Leishmania* vaccine.

## Materials and methods

### Ethics statement

The animal protocol for this study has been approved by the Institutional Animal Care and Use Committee at the Center for Biologics Evaluation and Research, Food and Drug Administration (ASP 1995#26). Further, the animal protocol is in full accordance with the “Guide for the Care and Use of Laboratory Animals” as described in the U.S. Public Health Service Policy on Humane Care and Use of Laboratory Animals 2015 **(http://grants.nih.gov/grants/olaw/references/phspolicylabanimals.pdf).**

### Animals and parasites

Five- to six-week-old female C57BL/6 mice were obtained from the National Cancer Institute, National Institutes of Health, Bethesda, MD. Five- to 6-week-old Lys-eGFP mice were obtained from NIH. 6- to 8-wk-old female mice were used for all the experiments. All mice were maintained in the Food and Drug Administration/CBER AAALAC-accredited facility under standard environmental conditions for this species. Both *LdWT* (MHOM/SD/62/1S) parasites and *LdCen^-/-^* line of *L*. *donovani* (Ld1S2D) used in our experiments were maintained in golden Syrian hamsters to maintain the infectivity. The parasites were cultured according to the procedure previously described [13,18]. Red fluorescent protein (RFP) expressing *LdWT* parasites were made using the pA2RFPhyg plasmid for the integration of an RFP/hygromycin B resistance gene expression cassette into the parasite’s 18S rRNA gene locus, as described previously [19]. *LdCen^-/-^* parasites expressing mCherry were generated using the pLEXSY-mCherry-sat2 plasmid, following the manufacturer’s protocol (Jena Bioscience). The parasites were cultured according to a procedure previously described [20].

### Neutrophil collection

Mice were injected with 3% thioglycolate (Sigma-Aldrich, Cat: T0632**)** and 5h after injection peritoneal exudate cells were obtained. The isolation of peritoneal neutrophils from peritoneal exudate cells was performed with the MiniMACS system (Miltenyi Biotech, Cat: 130-097-658). Neutrophil purity (92%) was confirmed by FACS prior to treatment or coculture with parasites. Neutrophils were plated in poly-L-Lysine–coated culture plate (Thermo Fisher Scientific, Cat: 356518).

### Transwell cell migration assay

Supernatant derived from uninfected, or neutrophils cultured under different treatment conditions were placed in the lower compartment of a transwell plate (96 well plate, 3.2mm diameter 5um pore size, ChemoTx System, NeuroProbe, UK). 10^5^ DCs were placed on top of the filter. Migration in medium alone acts as the negative control. After 3h of incubation at 37°C, 7 mM EDTA [prepared from 0.5M EDTA (Thermo Fisher Scientific, Cat: PR-V4231)] was added to the bottom wells for 10 min to release adhered cells from the well and filter. Cells from the lower chambers were then stained with trypan blue solution (Thermo Fisher Scientific, Cat: 15250061) and counted on a hemocytometer.

### Intradermal inoculation with *LdWT* or *LdCen*^-/-^parasites

C57BL/6 mice were infected through intradermal route in the ear with 10^6^ *LdWT* or *LdCen^-/-^* parasites or fluorescent *LdWT*^RFP^/ *LdCen*^*-/-* mCherry^ by means of a 31-gauge needle (EasyTouch U-100 Insulin Syringes MHC Medical Products, Cat: 831365) in a volume of 10 ml. The uninfected naive/control mice received 1x PBS (diluted from 10x PBS) (Quality biological, Cat: 119-069-131). To obtain chronically infected mice, C57BL/6 mice were infected 24 wk prior with 10^5^ *L*. *donovani* metacyclic promastigotes via tail vein. The CD4^+^ T cells were isolated from these mice using CD4 T cell isolation kit (Miltenyi Biotec, Cat: #130-104-454) and were used for coculture experiment with DCs at a 1:20 DC/CD4^+^ T cell ratio.

### Cultivation of BMDCs

Dendritic cells were cultured *in vitro* from bone marrow progenitors. Briefly, after sacrificing the mice their femurs and tibias were excised, cleaned of tissue, and flushed with RPMI medium (Thermo Fisher Scientific, Cat: 21875034**).** Erythrocytes were depleted by using ACK lysis buffer (Lonza, Cat: 10-548E) and isolated bone marrow was cultured with complete RPMI medium supplemented with 10% (v/v) fetal bovine serum (FBS) (R&D systems, Cat: S11150) and 1% penicillin (20 U/ml)/ streptomycin (20 μg/ml) (Thermo Fisher Scientific, Cat: 15140163) and 20 ng/mL GM-CSF (Peprotech, Cat: 315–03) and IL-4 (Peprotech, Cat: 214–14) for 7 days to obtain >75% purity of DCs (the purity of DCs were checked by CD11c-APC and mPDCA-1-FITC markers as per the Miltenyi Biotec, Cat: 130-100-875 and analyzed by flow cytometry). In a separate experiment, neutrophils were either remain uninfected or infected with *LdWT*/*LdCen^-/-^* parasites and were treated with LPS (1 μg/ml) (Sigma Aldrich Cat: L2630) for 8 h. The LPS from the neutrophil culture was rigorously washed out by repeated changes of media and co-cultured with isolated BMDCs for 24h. The conditioned media of BMDC cultures were assayed for mouse cytokine via sandwich ELISA. Culture supernatants were collected at 24h post infection to evaluate cytokine (IL-12) production with the use of sandwich ELISA kit (Thermo Fisher Scientific, Cat: 88-7121-22; Analytical sensitivity: 15 pg/mL; Assay range: 15–2,000 pg/mL). Costimulatory molecules expression on the surface of DCs were measured by flow cytometry where acquisitions of a million events were performed. The detailed method and antibody clones have been described in detail in the Flow cytometry section of the Materials and Methods.

### Parasite Load Determination by Quantitative PCR and Diff quick stain

The parasite load was determined in the infected neutrophils as previously described [21]. Briefly, infected neutrophils were lysed, and DNA was purified by using DNeasy Blood & Tissue kits (Qiagen). Seventy nanograms of sample DNA was used for template in a Taqman-based quantitative PCR. The target DNA was amplified from kinetoplast minicircle DNA of the parasite via using the following sequence of primers: *Leishmania* forward primer 5′- CTATTTTACACCAACCCCCAGT-3′ *Leishmania* reverse primer 5′-GGGTAGGGGCGTTCTGCGAAA-3′ along with the addition of a fluorescent probe 5′-RAAARKKVRTRCA GAAAYCCCGT-3′ for detection. A Black Hole Quencher moiety attached to the 3′ end and Calfluor Red was coupled to a C6 linker at the 5′ end. Fluorescent *Leishmania* probe (5′-RAAARKKVRTRCAGAAAYCCCGT-3′) was added in the reaction mixture at a final concentration of 1.5 pmols/μl. Cycling parameters were like this: preheat at 95°C for 180 s and then 40 two-step cycles of 95°C for 10 s and 55°C for 30 s. To measure the number of *Leishmania* cells that were represented by a given cycle threshold (Ct) value, a standard curve was made by purification of DNA from naïve mice neutrophils spiked with a known number of parasites.

For microscopic quantitation of parasite load, neutrophils were infected with the *LdWT/ LdCen^-/-^* parasites for 6 h at 37°C in 5% CO_2_ and washed with the medium, and then the cultures were incubated in RPMI medium for a 16h or 24h post-infection. The culture medium was removed, the slides were air dried and fixed via immersion in absolute methanol for 5 min at room temperature and stained using a Diff-Quick stain set (Baxter Healthcare Corp., Miami, FL), and intracellular parasite numbers were evaluated microscopically. To measure parasite load in these cultures, a minimum of 300 neutrophils were counted. The results are expressed as percentages of neutrophils that were infected by parasites.

### Neutrophil depletion

Neutrophil depletion was carried out by i.p. injection of *in vivo* mAb anti-mouse Ly6G (1A8) (1 mg) (BioXCell, Cat: BE0075-1) or GL113 (*in vivo* mAb IgG2a isotype control; BioXCell, Cat: BE0089) 1 d prior to parasite injection and every 48 h after i.d. injection with the parasites till day 5. At day 5, the efficiency and specificity of the depletions were evaluated on lymph node cell preparation as previously described [9,22].

### CCL3 depletion:

1. In neutrophil supernatants; Neutrophil supernatant were placed on a plate coated with polyclonal antibody from the CCL3 ELISA kit (R&D systems, Cat: MMA00). Depletion in supernatant was validated by ELISA. 2. *in vivo* depletion; anti-mouse CCL3 nAbs were obtained from R&D Systems, Cat: AF-450-NA) and reconstituted in water for injections. After reconstitution, the nAb 4 μg/5 μl was singly administered *i*.*p*. to mice on day 1 prior to parasite injection. As controls, mice were injected with a similar regimen of PBS.

### Processing of ear dLN and parasite burden estimation

Retro maxillary (ear draining) lymph nodes were removed and mechanically dissociated using tweezers and a syringe plunger. Tissue homogenates were filtered through a 70-mm cell strainer (Falcon Products). Parasite burden in the ear draining lymph nodes (dLN) were measured by limiting dilution analysis as previously described [23]. Parasite titrations in the ear dLNs were done via serial dilution (1:1 dilutions) of tissue homogenates in 96-well flat-bottom microtiter plates (Corning, NY) in M199 in triplicate and incubated at 26°C without CO_2_ for 7–10 days. The greatest dilution yielding viable parasites was noted and data are presented the mean parasite dilution ± SEM.

### Infection of mice and isolation of parasitized neutrophils, and DCs

The mice were injected intradermally in the ear pinna either with PBS (uninfected naïve mice) or 10^6^ stationary-phase *LdWT/LdCen^-/-^* promastigotes. In each study, a minimum of six mice were used per group. At 5d post-infection, mice were sacrificed and parasitized neutrophils (Cd11b^+^ Ly6C^int^ Ly6G^+^RFP/mCherry^+^) and parasitized DCs (Cd11b^+^Cd11c^+^ MHCII^hi^ RFP/mCherry^+^) total DCs (Cd11b^+^Cd11c^+^Ly6G^-^Ly6C^-^MHCII^hi^) from different groups of mice were sort selected using the BD FACS Aria-II. In some experiments, Lys- eGFP mice were infected with *LdWT^RFP^/ LdCen^-/- mCherry^* parasites. At 5d post-infection, Cd11b^+^Cd11c^+^MHCII^hi^eGFP^hi^RFP^+^ (hereafter called P1) and Cd11b^+^Cd11c^+^MHCII^hi^eGFP^-^RFP^+^ (hereafter called P2) from different groups of mice were sort selected. Single-cell suspensions were prepared from ear dLN, then labeled for fluorochrome tagged anti–TCR-b, anti-NK1.1, anti-Cd19 Abs using fluorochrome tagged magnetic beads, and passed through LS columns to select out these cell types. Flow through–enriched population was collected and stained with neutrophil and DC-specific markers and further sort selected. Fluorescence minus one control was used for proper gating of positive events for designated fluorophores. The post sort analysis was performed after each sort. The sorted neutrophils were placed on 8 well Nunc chambered cover glass and counterstained with Hoechst 33342 (2 μg/ml). The cells were visualized using Leica SP8 confocal microscope with excitation 561 and 405 nm for red and blue channel imaging respectively. In some experiments, the isolated parasitized neutrophils and DCs were visualized using Leica SP8 confocal microscope. Briefly, the sorted and labelled cells were placed on ibidi slide. Excitation 670, 590 and 405 nm was used for alexa700, mRFP and Pacific blue channels imaging respectively. Scientific Volume Imaging Huygens professional and Bitplane Imaris software were used for deconvolution and 3D visualization for all the experiments.

### Neutrophil elastase activity assay

The sort selected P1 and P2 DCs were adhered on polylysine (0.01%)-treated coverslips in serum-free medium for 2 h at 37°C. The neutrophil elastase (NE) activity was measured by mouse neutrophil elastase ELISA Kit (Abcam, Cat: ab252356; Sensitivity: 27.39 pg/ml; Range: 62.5 pg/ml—4000 pg/ml) as per manufacture’s protocol.

### Chemokine ELISA

Chemokine (CCL3) concentration in the culture supernatant of LPS treated infected/uninfected neutrophils was quantified using ELISA kit (Abcam, Cat: ab 200017; Sensitivity: 1.95 pg/ml, Range: 15.6 pg/ml—1000 pg/ml). The assay was performed according to the manufacturer’s instructions.

### *Ex vivo* DC and T cell coculture studies

P1 and P2 DCs or parasitized DCs were flow sorted from ear dLN 5d post infection. These were cocultured *in vitro* with CD4^+^ T cells (at a 1:20 DC/CD4^+^ T cell ratio) isolated and purified from 24-*wk L*. *donovani–*exposed mice. After 5 d of incubation in a 5% CO2 humidified chamber at 37°C, T cell proliferation was measured by studying the dilution of CFSE (Thermo Fisher Scientific, Cat: 34554**)** in CD4 stained CD44^hi^ T cells via flow cytometry.

### RT PCR

Total RNA was extracted from 1) thioglycolate-elicited peritoneal neutrophils, 2) parasitized neutrophils/ DCs recruited in ear dLN following ID injection of either PBS/*LdWT/LdCen^-/-^* parasites 3) P1 and P2 DCs sort selected from ear dLN following ID injection of either PBS/*LdWT/LdCen^-/-^* parasites in Lys-eGFP mice 4) CD4^+^T cells purified from ear dLN following ID injection of either PBS/*LdWT/LdCen^-/-^* parasites 5) Total ear dLN lymph nodes were purified by using an RNAqueous-Micro Kit (AM1931; Ambion), which also eliminates any contaminating DNA by using on-column PureLink DNase treatment during RNA purification. Four hundred nanograms of total RNA was reverse transcribed into cDNA by using random hexamers with a high-capacity cDNA reverse-transcription kit (Applied Biosytems). Cytokine-chemokine gene expression levels were determined using the TaqMan Gene Expression Master Mix and premade TaqMan gene expression assays (Applied Biosystems) using a CFX96 Touch Real-Time System (Bio-Rad Laboratories, Hercules, CA). The data were analyzed with CFX Manager Software. Expression of the following genes was determined using TaqMan gene expression assays (Applied Biosystems) in the CFX96 Touch Real-Time System: CCL3 (Mm99999057_m1), CCL4 (Mm00443111_m1), CCL5 (Mm01302427_m1), IL-12 (Mm00434174_m1), TNF-α (Mm00443258_m1), IL-10 (Mm00439614_m1), Tbet (Mm00450960_m1), GAPDH (Mm99999915_g1). Expression values were determined by the 2- ΔΔ Cycle threshold method. Samples were normalized to GAPDH expression and determined relative to expression values from untreated samples or PBS-injected mice as appropriate.

### Flow cytometry

The ear dLNs were removed from animals, and single-cell suspension was prepared. For surface staining, cells were blocked via using rat anti-mouse CD16/32 (Clone: 2.4G2, BD Biosciences, Cat: 553141) at 4°C (5 mg/ml) from BD Pharmingen for 20 min. Cells were then stained with anti-mouse Ly6G (Clone: 1A8-Ly6g, Thermo Fisher Scientific, Cat: 25-9668-82), anti-mouse CD11b (Clone: M1/70, Thermo Fisher Scientific, Cat: 56-0112-82), **anti-mouse Ly6C (**Clone: HK1.4, Biolegend, Cat: 128006), anti-mouse CD3 (Clone: eBio500A2 (500A2), Thermo Fisher Scientific, Cat: 56-0033-82), anti-mouse CD4 (Clone: RM4-5, Bio legend, Cat: 100552), anti-mouse CD44 (Clone: IM7, Thermo Fisher Scientific, Cat: 48-0441-82), anti-mouse Cd11c (Clone: N418, Thermo Fisher Scientific, Cat: 48-0114-80), anti-mouse Ly6G/Ly6C(Gr-1) (Clone: RB6-8C5, Biolegend, Cat: 108416), anti-mouse CD80 (Clone 16-10A1, BD), anti-mouse MHCII Class II (I-A/I-E) (Clone: M5/114.15.2, Thermo Fisher Scientific, Cat: 47-5321-82), anti-mouse CD40 (Clone: 1C10, Thermo Fisher Scientific, Cat: 17-0401-82), anti-mouse CD205 (Clone: 205yekta, Thermo Fisher Scientific, Cat:25-2051-42), anti-mouse CD8a Antibody (Clone: 53–6.7, Bio legend, Cat: 100711) (each with 1:100 dilution; at 4°C). For MPO staining, cells were stained with MPO Polyclonal Antibody (Clone: bs-4943R, Thermo Fisher Scientific, Cat: BS-4943R) at 1:50 dilution in blocking buffer and incubated for 30 min at room temperature, washed twice with 2%BSA in PBS, followed by secondary antibody incubation for 40 min at room temperature. In order to stain the dead cells for all the flow experiments, the samples were stained with Live/Dead Fixable Aqua (Thermo Fisher Scientific, Cat: L34957).

Cells were then washed twice with wash buffer [1x PBS, 2–5% (v/v) FBS (or BSA) 2 mM EDTA] followed by fixation with a Fixation/Permeabilization Solution Kit (BD Bioscience, Cat: 554714) for 20 min at room temperature and acquired on an LSR II (BD Biosciences) equipped with 407-, 488-, 532-, and 633- nm laser lines using FACS Diva 6.1.2 software. Acquisitions of a million events were performed. Data were analyzed with FlowJo software version 9.7.5 (Tree Star). For analysis, first doublets were removed using width parameter; dead cells were excluded based on staining with the Live/Dead Aqua dye. Lymphocytes were identified according to their light-scattering properties. CD4 T cells were identified as CD3^+^ lymphocytes uniquely expressing CD4.

### Statistical analysis

Statistical analysis of differences between means of groups was determined either by unpaired two-tailed Student t test or one- way ANOVA (with a post-test correction for type II error) or two-way ANOVA, using GraphPad Prism 5.0 software. A p value < 0.05 was considered significant, and a p value <0.005 was considered highly significant.

## Result

### 1. *LdCen^-/-^* phagocytized neutrophils produce increased CCL3 chemokine and recruit higher number of bone marrow-derived DCs compared to *LdWT* infection *in vitro*

**Fig 1:**
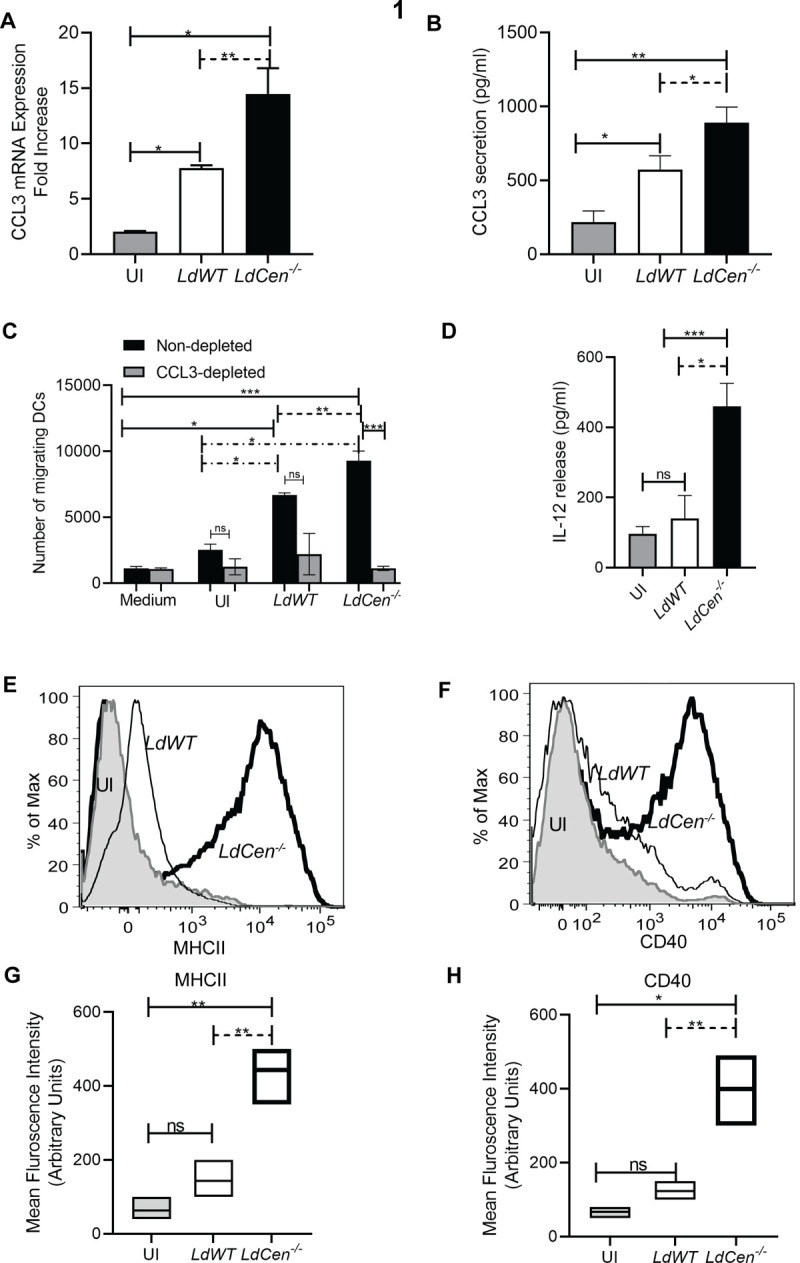
In presence of *LdCen^-/-^*, neutrophils produce increased CCL3 which chemo-attracts higher bone marrow-derived DCs compared to *LdWT* infection *in vitro*. **(A)** Peritoneal neutrophils were either left uninfected or infected with *LdWT/LdCen^-/-^* parasites for 16h. Changes in the mRNA expression levels of CCL3 from uninfected and infected neutrophils were determined by qPCR as described in Materials and Methods. The data represent the mean values ± SD of results from three independent experiments that all yielded similar results. **(B)** 24 hours after infection, the levels of CCL3 in the culture supernatant were evaluated by sandwich ELISA as described in Materials and Methods. ELISA data are expressed as means ± SD of values from triplicate experiments that yielded similar results. *p < 0.05; **, p < 0.005. **(C)** Absolute number of dendritic cells that migrated after stimulation with supernatants from uninfected peritoneal neutrophils or neutrophils cultured in presence of *LdWT* or *LdCen^-/-^* infection under CCL3 non-depleted or depleted condition was measured by chemotaxis assay. The data represent the mean values ± SD of results from three independent experiments that all yielded similar results. *p< 0.05, ** p< 0.005, *** p < 0.0005 between the groups. **(D)** BMDCs were cocultured with either LPS treated uninfected or infected neutrophils as described in Materials and Methods. Culture supernatants were collected to determine the IL-12 release by ELISA. The data represent the mean values ± SD of results from 3 independent experiments that all yielded similar results. * p < 0.05; *** p < 0.0005 between the groups. **(E, F)** Layouts and **(G, H)** Mean fluorescence intensity (MFI) of MHCII and CD40 in uninfected and *LdWT* or *LdCen^-/-^* infected neutrophil-BMDC coculture. The data represent the mean values ± SD of results from 3 independent experiments that all yielded similar results. * p < 0.05; ** p < 0.005 between the groups.

Neutrophil derived chemokine CCL3 plays a major role in DC recruitment in *L*. *major* infection in mice [8]. We therefore investigated its expressions in neutrophils in response to *LdCen^-/-^* infection *in vitro* and compared with *LdWT* infection. Infection of neutrophils with either *LdWT* or *LdCen^-/-^* parasites resulted in an increase in CCL3 mRNA level by an average of 3-fold and 7-fold over uninfected neutrophils respectively (Fig 1A). Next, we evaluated CCL3 protein levels in culture supernatants of uninfected and infected neutrophils 24h after infection. Infection with *LdWT* or *LdCen^-/-^* parasites led to a significant induction of CCL3 secretion from infected neutrophils compared to uninfected neutrophils (Fig 1B). Importantly, production of CCL3 in *LdCen^-/-^* parasite-infected neutrophils was ~2-fold higher than in *LdWT* infected neutrophils (Fig 1B). RT-PCR assay to measure the *Leishmania* minicircle DNA and microscopic analysis to determine the percentage of infected neutrophils showed a comparable *LdWT/ LdCen^-/-^* parasite infection in the neutrophils at 16 and 24 h time points following infection (S1A–S1D Fig) suggesting that the observed differences in CCL3 levels between *LdWT* and *LdCen*^−/−^ as reported in Fig 1A and 1B, are not due to variation in the level of parasite infection. We also determined the mRNA levels of other DC-attracting chemokines such as CCL4 and CCL5 in the neutrophils infected with *LdWT* or *LdCen^-/-^* parasites. There was no significant difference in the expression of CCL4 (S1E Fig) and CCL5 (S1F Fig) between *LdWT* and *LdCen^-/-^* infections, and both were higher than uninfected controls.

We next assessed the effect of chemokines in the supernatants of uninfected or *LdWT/ LdCen^-/-^* infected neutrophils on chemoattraction of DCs using Transwell cell migration assays. Bone marrow-derived DCs were deposited on the filter of a Transwell migration assay plate, the lower compartment containing the supernatants recovered either from uninfected neutrophils or neutrophils exposed to either *LdWT or LdCen^-/-^* parasites. DCs exposed to supernatants from *LdWT* or *LdCen^-/-^* infected neutrophils showed significantly higher migration compared to uninfected or media alone (Fig 1C). Consistent with the elevated levels of CCL3 observed in *LdCen*^-/-^ infected neutrophils, a significant increase of DC migration was observed towards supernatants from *LdCen^-/-^* infected neutrophils compared to that from *LdWT* infection (Fig 1C). Since we had observed production of higher levels of CCL3 and not the CCL4 and CCL5 chemokines by *LdCen^-/ -^*infected neutrophils, we therefore assessed whether chemo-attractive activity of CCL3 itself is necessary and sufficient to explain the elevated DC migration observed in *LdCen*^-/-^ infection. To test this hypothesis, CCL3 was depleted from the supernatant of uninfected or infected neutrophils. The CCL3 depleted supernatants were analyzed for their chemo-attractive activity for DCs using the Transwell migration assay. The number of DCs that migrated towards supernatants of CCL3 depleted *LdWT* infected neutrophils, was comparatively lower although statistically not significant compared to supernatants of untreated *LdWT* infected cells (Fig 1C). In contrast, we found a significant reduction in DC migration towards CCL3 depleted *LdCen^-/-^* infected neutrophils than those that migrated to supernatants of untreated *LdCen^-/-^* infected cells (Fig 1C). These data show that neutrophil derived CCL3 plays a major role in the migration of DCs specifically during *LdCen^-/-^* infection *in vitro*.

Next, we investigated whether *LdWT* or *LdCen^-/-^* infected neutrophils trigger production of cytokines and expression of costimulatory molecules upon interactions with DCs *in vitro*. We observed *LdCen^−/−^* parasite infected, but not *LdWT* infected neutrophils significantly induced the production of IL-12 by DCs (Fig 1D). Further, we analyzed the expression of costimulatory molecules after interaction of parasite infected neutrophils with DCs. Flow-cytometric analysis showed that compared to *LdWT* infected neutrophils, *LdCen^−/−^* infected neutrophils induced significantly higher levels (both MFI and % positivity) of MHCII (Figs 1E, 1G and S1G) and CD40 (Figs 1F, 1H and S1G) expression on DCs.

### 2. *LdCen^-/-^* phagocytized neutrophils produce increased CCL3 chemokine and recruit higher number of dendritic cells to the ear dLN of mice compared to *LdWT* infection

**Fig 2:**
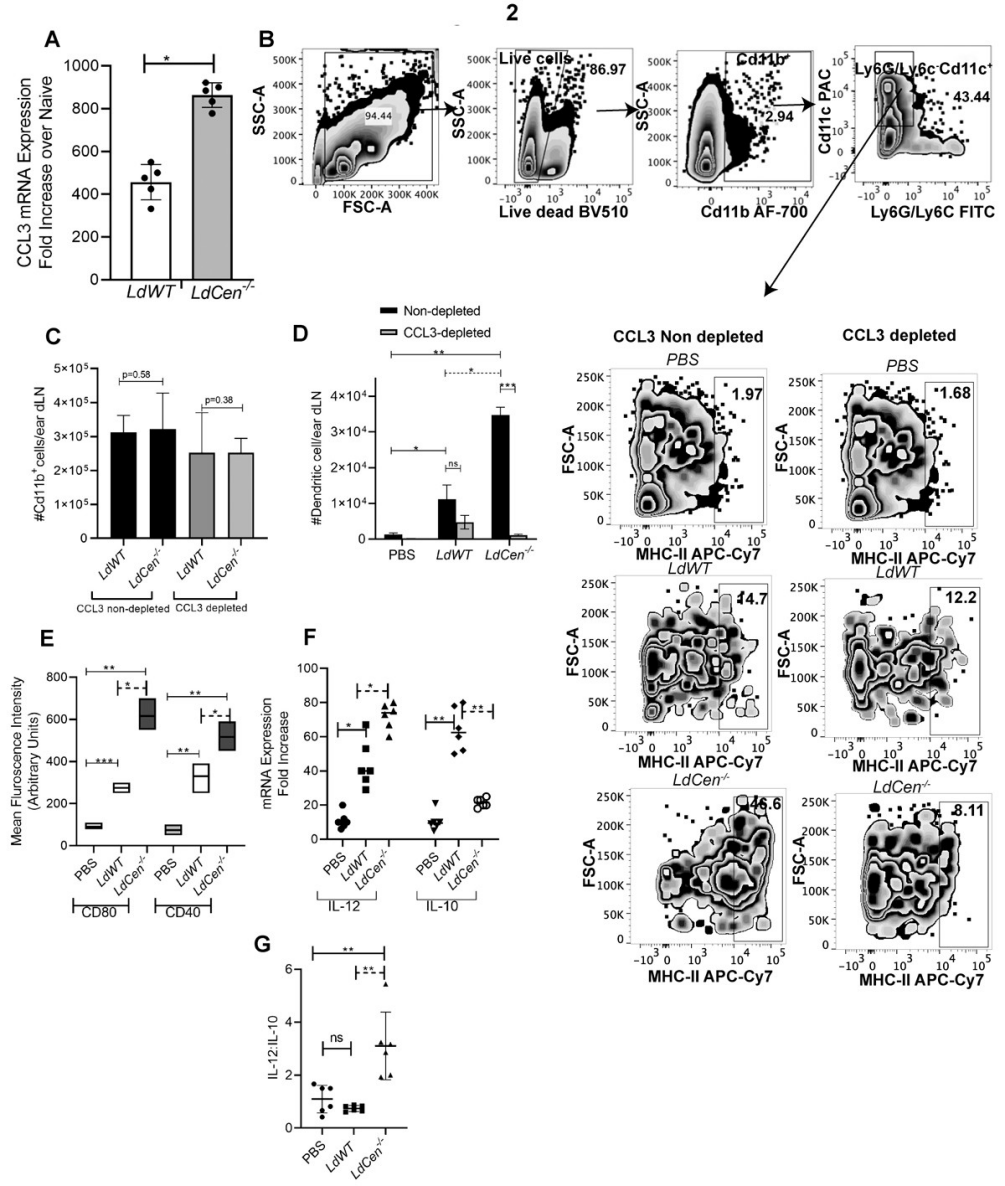
During *LdCen^-/-^* ID infection, neutrophil derived increased CCL3 recruits significantly higher number of dendritic cells attributed with proinflammatory phenotype in the ear dLN compared to *LdWT* infection. **(A)** Parasitized neutrophils (Cd11b^+^Ly6G^+^ Ly6c^int^RFP/mCherry^+^) were flow sorted from the ear dLN 5d post infection and mRNA expression levels of CCL3 from sorted neutrophils in the ear dLN were estimated by qPCR as described in Materials and Methods and expressed as fold change from uninfected naive mice. The experiment was repeated three times with pooled digests from five to six ear dLNs per experiment. The data represent the mean values ± SD of results from three independent experiments that all yielded similar results (n = 5). ** p < 0.005 between the groups. **(B)** The gating strategy and individual flow plots for DCs (CD11b^+^ Ly6G^**−**^Ly6C^**−**^ CD11c^**+**^MHCII^hi^) recruitment in ear dLNs 5d post infection is shown under CCL3 non-depleted or depleted condition. **(C**) Total number of Cd11b^+^ cells recruited in ear dLN 5 days post infection in mice depleted or not of CCL3 has been shown. **(D)** Changes in the total number of DCs per ear dLN. Values shown are the mean numbers of cells per ear dLN ± SD of results. 6–8 ear dLN, pooled data from 3 independent experiments that all yielded similar results (n = 6). *p< 0.05; ** p < 0.005, *** p < 0.0005 between the groups. (**E)** Parasitized DCs (CD11b^+^CD11c^**+**^MHCII^hi^RFP/mCherry^+^) were flow sorted from the ear dLNs 5d post infection. The Mean fluorescence intensity (MFI) of CD80 and CD40 in flow sorted parasitized DCs from ear dLN were calculated based on flow cytometry results. The data represent the mean values ± SD of results from 3 independent experiments that all yielded similar results (n = 6). * p < 0.05; ** p < 0.005; *** p < 0.0005 between the groups. **(F)** Changes in the expression of IL-12 and IL-10 mRNA in sort-selected parasitized DCs were determined by qPCR as described in Materials and Methods **(G)** and their ratio was determined. The data represent the mean values ± SD of results from three independent experiments that all yielded similar results (n = 6). *p< 0.05; ** p < 0.005; between the groups.

Next, we studied how neutrophil derived CCL3 influences DC recruitment *in vivo* to ear dLNs of C57BL/6 mice following ID injection with PBS or *LdWT*or *LdCen*^-/-^ parasites 5d post infection. Parasitized neutrophils were sort selected from ear dLN of either *LdWT ^RFP^* or *LdCen*^*-/-* mCherry^ parasite injected mice by gating on live single Cd11b^+^Ly6C^int^Ly6G^+^*RFP/mCherry^+^* cells (S2A–S2E Fig) and assessed the expression profiles of chemokines CCL3, CCL4 and CCL5. Neutrophil’s sort selected from *LdCen^-/-^* infected mice ear dLNs showed a significant increase in the expression of CCL3 compared to *LdWT* infected mice (Fig 2A). There was no difference in the expression of other chemokines CCL4 (S2F Fig) and CCL5 (S2G Fig) in these infections.

We next confirmed the specificity of neutrophil derived CCL3 in the recruitment of DCs to ear dLN via depleting CCL3 *in vivo* using a CCL3 nAb followed by ID injection with PBS or *LdWT* or *LdCen^-/-^* parasites. The gating strategy and individual flow plots are shown in Fig 2B. Nearly equal number of total CD11b^+^ cells were found in *LdWT* and *LdCen^-/-^* infected mice ear dLNs, albeit the overall number of CD11b^+^cells were comparatively lower in CCL3 depleted infected mice compared to non-depleted condition (Fig 2C). Interestingly, CCL3 depletion significantly reduced the number of DCs (Cd11b^+^Cd11c^+^Ly6GLy6C^-^MHCII^hi^) in ear dLN of *LdCen^-/-^* infected mice whereas no significant reduction was observed in *LdWT* infected mice after CCL3 depletion as shown by the gating strategy and individual flow plots (Fig 2B) and subsequent quantitative representation showing the DC numbers (Fig 2D). Further using additional markers for DCs, we found that CCL3 depletion attenuated the recruitment of another subtype of DCs (Cd11b^+^Cd11c^+^MHCII^hi^CD8a^-^CD205^low^) in ear dLN of *LdCen^-/-^* infected mice whereas no significant reduction was observed in *LdWT* infected mice after CCL3 depletion (S2H–S2I Fig).

It is possible that DCs may arrive at the site of infection by homeostatic migration in addition to active recruitment by chemokine gradients. We therefore characterized the phenotype of DCs recruited/migrated to the ear dLN in *LdCen^-/-^* infected mice and compared with *LdWT* infected mice. Parasitized DCs were sort selected from different groups of mice ear dLNs by gating live single cells (Cd11b^+^Cd11c^+^ MHCII^hi^ RFP/mCherry^+^) (S2J–S2L Fig) and assessed for the expression of costimulatory molecules and cytokines. Flow-cytometric analysis of parasitized DCs from *LdCen^-/-^* group ear dLN showed a significantly higher level of CD80 and CD40 expression (Fig 2E) and produced significantly higher IL-12 along with a concomitant decrease of IL10 (Fig 2F) with an overall higher IL12:IL-10 ratio compared with *LdWT* group (Fig 2G).

### 3. Dendritic cells in ear dLN are predominantly infected by phagocytosing infected neutrophils

**Fig 3:**
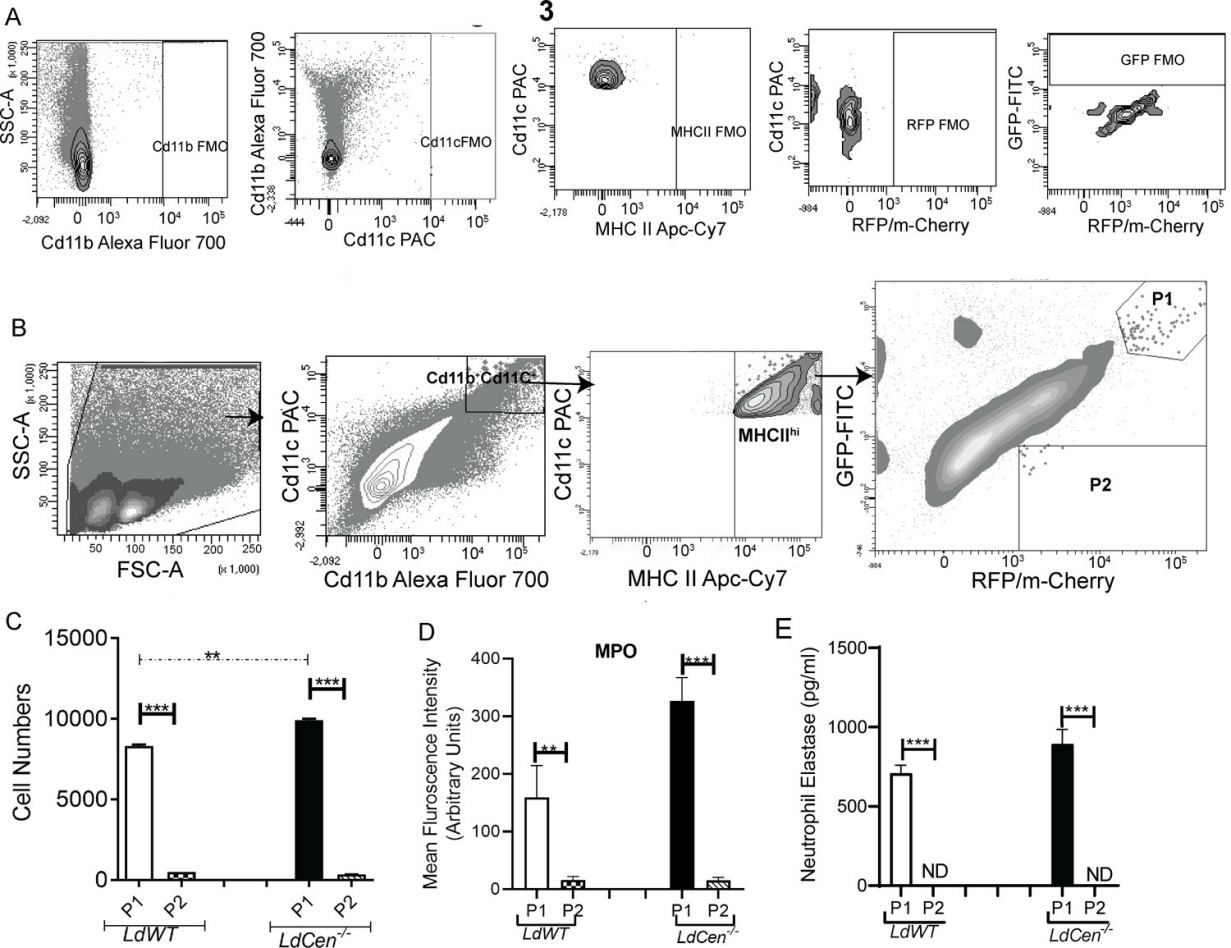
Dendritic cells in ear dLN predominantly become infected by phagocytosing infected neutrophils 5d post infection and express neutrophil markers. **(A)**The FMO’s for all the fluorophores used in (B) have been shown and are based on the same gating as in (B). **(B)** Cd11b^**+**^Cd11c^+^MHCII^hi^ GFP^hi^RFP^+^ (P1) and Cd11b^**+**^ Cd11c^+^ MHCII^hi^ GFP^-^RFP^+^ (P2) dendritic cells were flow sorted from the ear dLN of LYS-eGFP mice 5d post infection with either *LdWT* or *LdCen^-/-^* parasites. The sorting strategy has been displayed (n = 6). **(C)** Changes in the total number of P1 and P2 DCs per ear dLN. Values shown are the mean numbers of cells per ear dLN ± SD of results. 6 ear dLN, pooled data from 3 independent experiments that all yielded similar results (n = 6). * p < 0.05 **(D)** Mean fluorescence intensity of MPO in P1 and P2 DCs sort selected from infected LYS-eGFP mice 5d post infection. **(E)** The sort selected P1 and P2 DC population were assayed for NE activity as described in Materials and Methods. The NE activity was expressed as the absorbance observed at 450 nm. Data are pooled from three independent repeats and are shown as means ± SD that all yielded similar results (n = 6). *** p < 0.0005 between the groups.

To study the interaction and uptake of neutrophils by DCs *in vivo*, fluorescent *LdWT*^RFP^ or *LdCen*^*-/-* mCherry^ parasites were injected into the ear dermis of LYS-eGFP mice which enables identification of neutrophils by GFP expression. We observed two types of DCs from the ear dLN after sorting; viz; Cd11b^+^Cd11c^+^MHCII^hi^eGFP^hi^RFP^+^ (hereafter called P1) and Cd11b^+^Cd11c^+^MHCII^hi^eGFP^-^RFP^+^ (hereafter called P2). The sorting strategies are shown in Fig 3A and 3B. The number of P1 DCs were significantly higher than P2 DCs in both *LdWT* and *LdCen^-/-^* infected mice ear dLNs thereby suggesting that the majority of the DCs acquired the parasites via uptake of infected neutrophils (Fig 3C). The number of P1 DCs was significantly higher in *LdCen^-/-^* infected mice ear dLN compared with *LdWT* infected mice (Fig 3C). To confirm that P1 DCs acquired the parasites by engulfing neutrophils, whereas P2 DCs acquired the parasites directly without neutrophil involvement, the sorted P1 and P2 DCs were either stained for neutrophil specific marker neutrophil derived-myeloperoxidase (MPO) or assessed for enzymatic activity of neutrophil elastase (NE). The MPO expression was predominant in sort selected P1 DCs in both *LdWT* and *LdCen^-/-^* infected mice whereas P2 DCs showed negligible expression of MPO (Fig 3D). Likewise, P1 DCs from infected mice exhibited heightened NE enzymatic activity in the culture supernatant whereas NE enzymatic activity was undetectable in the culture supernatants of P2 DCs from infected mice (Fig 3E). These results indicate that following *LdWT* or *LdCen^-/-^* infections, DCs acquire parasites predominantly by phagocytosing parasitized neutrophils rather than direct neutrophil-independent mechanisms.

### 4. Higher expression of proinflammatory cytokines, co-stimulatory molecules and antigen presentation in P1 DCs

**Fig 4:**
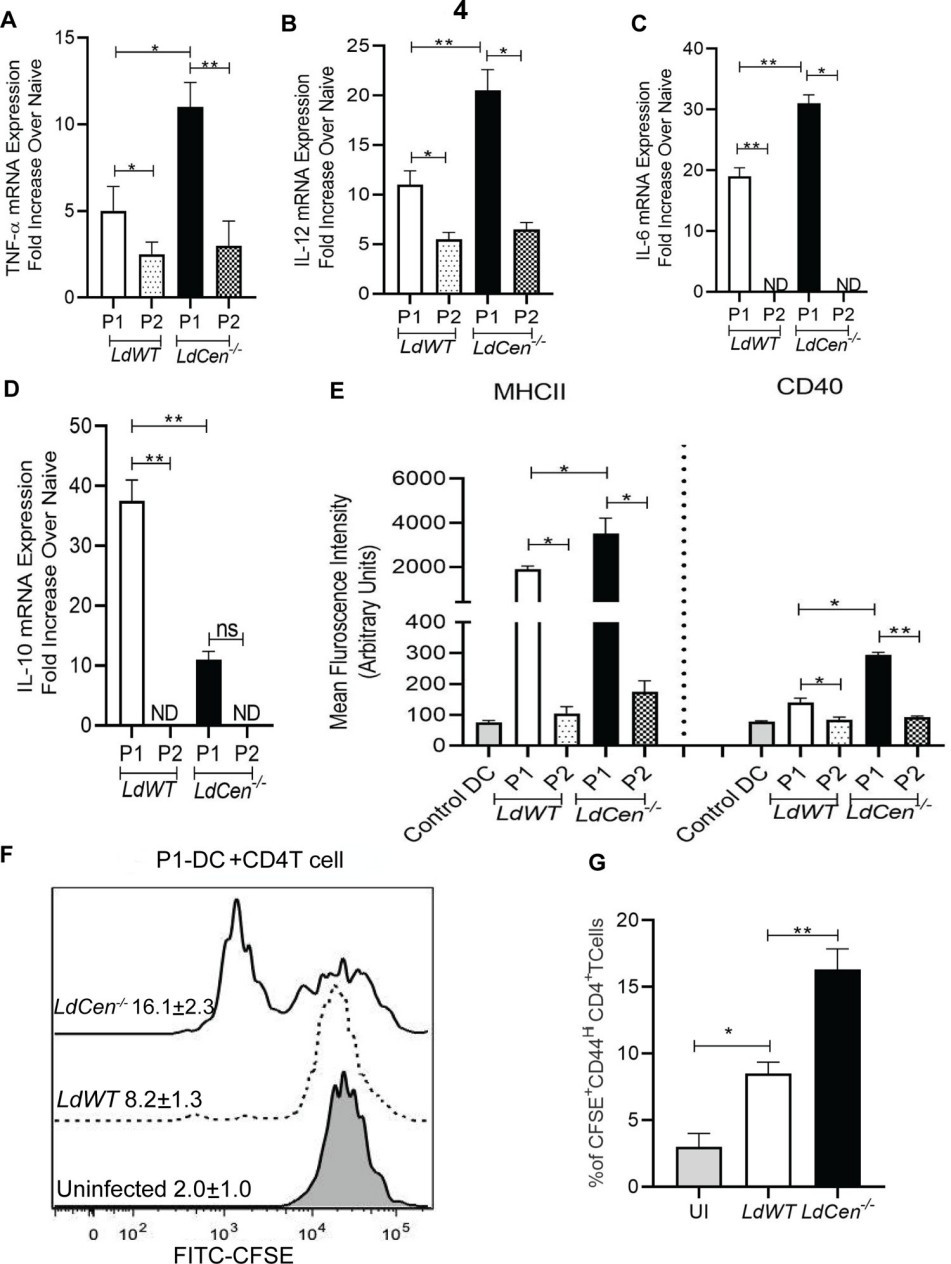
Higher expression of proinflammatory cytokines, co-stimulatory molecules and subsequent antigen presentation in P1 DCs that have captured *LdCen^-/-^* infected neutrophils compared to P1 DCs containing *LdWT* infected neutrophils. The P1 and P2 DC population from *LdWT* and *LdCen^-/-^* infected LYS-eGFP mice ear dLN were flow sorted as described in Fig 3 legend. Normalized expression levels of cytokines, such as **(A)** TNF-α (**B)** IL-12 **(C)** IL-6 and **(D)** IL-10 were estimated. Data are presented as fold change from uninfected naive mice. The data represent the mean values ± standard deviations of results from 3 independent experiments that all yielded similar results (n = 6). * p < 0.05; **p< 0.005. **(E)** The Mean fluorescence intensity (MFI) of the costimulatory molecules (MHCII and CD40) expression on P1 and P2 DCs from *LdWT/LdCen^-/-^* infected mice or in Cd11C^+^ DCs from uninfected mice (control mice) ear dLN was studied and represented by the bar diagram. The data represent the mean values ± SD of results from three independent experiments that all yielded similar results (n = 6). * p < 0.05; **p< 0.005. (**F, G**) Ag-specific CD4 T cell proliferation was estimated from P1-DC-CD4T cell coculture assay by studying CFSE dilution of gated CD4^+^CD44^+^ T cells and is represented by the staggered offset histogram overlay and bar diagram. Cell proliferation was analyzed in triplicate experiments (n = 6), and histograms representative of mean values were overlaid for the figure.

We next characterized the phenotype of P1 and P2 DCs recruited to the ear dLNs in *LdCen^-/-^* infected mice and compared with *LdWT* infected mice. Sort selected P1 and P2 DCs from ear dLNs of mice infected with *LdWT* or *LdCen^-/-^* parasites were assessed for their phenotype. RT-PCR analysis showed proinflammatory cytokines, viz; TNF-α (Fig 4A), IL-12 (Fig 4B) and IL-6 (Fig 4C) were significantly elevated in P1 DCs isolated from *LdCen^-/-^* infected mice compared with *LdWT* infected mice. In contrast, IL-10, the anti-inflammatory cytokine was significantly reduced in P1 DCs isolated from *LdCen^-/-^* infected mice compared with *LdWT* infected mice (Fig 4D). Overall, the expression of all the cytokines in P2 DCs was either lower than the P1 DCs or undetectable and not statistically different between *LdWT* and *LdCen^-/-^* infected mice (Fig 4A–4D). The RNA expression data of few of the cytokines could not be detected due to the lower yield of P2 population using stringent sorting strategy.

To test the P1/P2 DC mediated T cell activation, we first assessed the expression of costimulatory molecules in the P1 and P2 DCs recruited to the ear dLNs of C57BL/6 mice following ID injection with PBS /*LdWT* / *LdCen^-/-^* parasites 5d post infection. P1 DCs from *LdCen^-/-^*infected mice showed a significantly higher level of MHCII and CD40 expression compared with *LdWT* (Figs S3A and 4E). However, the expression of costimulatory molecules in P2 DCs was significantly lower than P1 DCs in both groups of infected mice (Figs S3A and 4E).

To test the antigen presentation capability of P1 and P2 DCs, we sort selected P1 and P2 DCs from ear dLNs of *LdWT^RFP^* / *LdCen^-/-mCherry^* infected mice 5d post-infection. The sort-selected DCs were cultured with CFSE-labeled CD4^+^ T cells isolated from the spleens of mice previously infected with *L*. *donovani* and recovered from infection. Following 5 days of coculture, P1 DCs from *LdCen^-/-mCherry^* infected mice induced a significant increase in Ag-specific CD4^+^T cell proliferation compared to those cocultured with P1DCs from *LdWT* infected mice as shown by the offset histogram depicting the individual percentage of proliferating CD4^+^T cells from different groups (Fig 4F) and the quantitative bar diagram indicating the percentage of cells showing CFSE dilution on CD44^+^CD4^+^ gated cells (Fig 4G). Notably, negligible T cell proliferation was observed in the infected P2-DC from either *LdWT* or *LdCen^-/-^* infected mice and CD4 T cell cocultures (S3B Fig).

### 5. Neutrophil depletion abrogates recruited/ migrated DC numbers in ear dLN and antigen presenting capacity of DCs in *LdCen^-/-^* immunized mice

**Fig 5:**
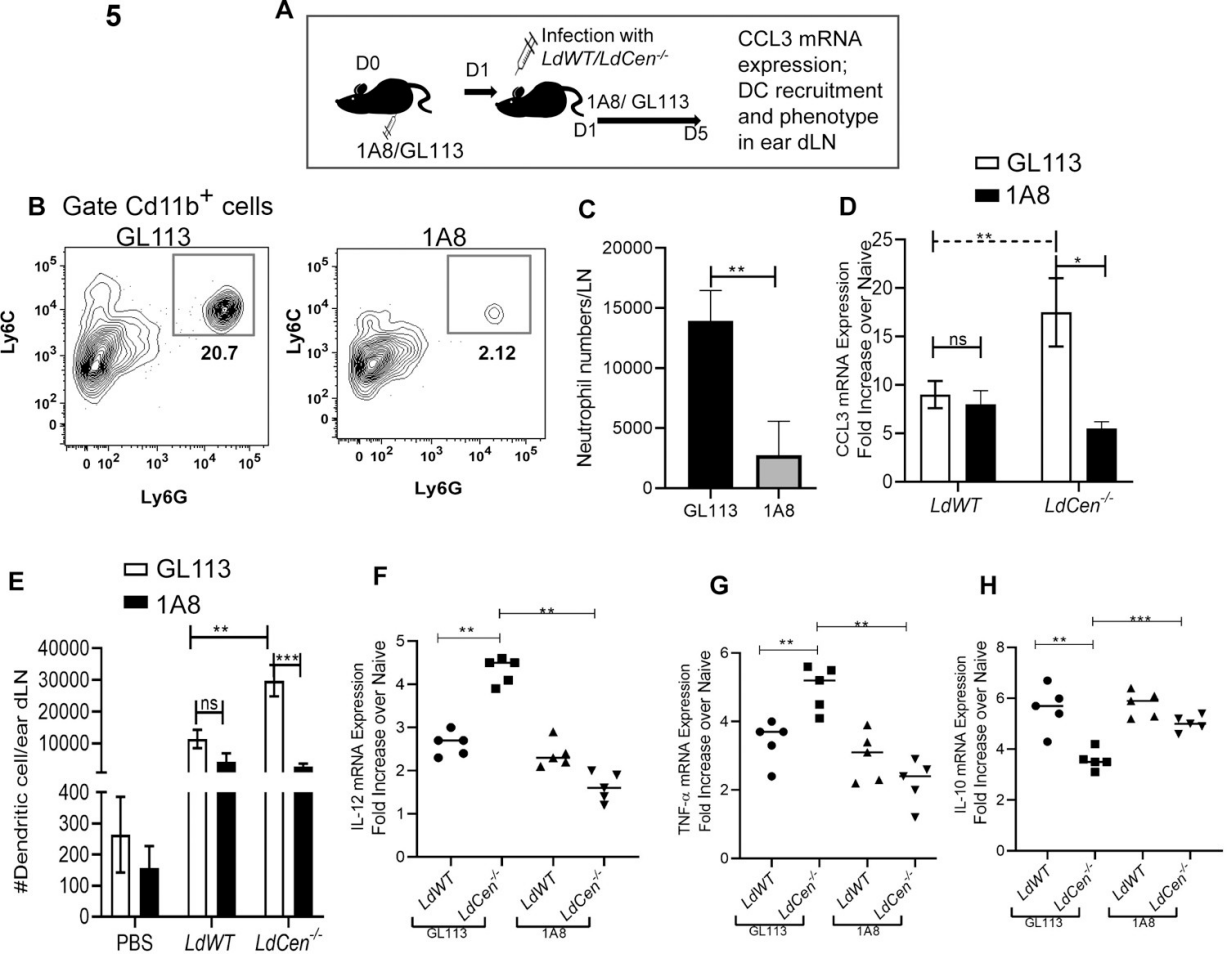
Neutrophil depletion abrogates CCL3 generation, DC recruitment in *LdCen^-/-^* infected mice ear dLN: recruited DC’s exhibit anti-inflammatory phenotype. **(A)** Schematic diagram showing experimental scheme for neutrophil depletion and subsequent experiments. (**B, C**) Representative plots and bar diagram showing mean total number of neutrophils per lymph node on day 5 in the GL113 or 1A8 treated mice. Data are pooled from three independent repeats and are shown as means ± SD that all yielded similar results (n = 6) ** p < 0.005. **(D)** RT-PCR analysis of total RNA isolated from draining lymph nodes of GL113/1A8 treated infected mice at 5d post infection as described in Materials and Methods. Normalized expression levels of CCL3 were estimated. Data are presented as fold change from uninfected naive mice. The data represent the mean values ± SD of results from 3 independent experiments that all yielded similar results (n = 6). ** p< 0.005. **(E)** Changes in the total number of DCs per ear dLN. Values shown are the mean numbers of cells per ear dLN ± SD of results. Six ear dLN and pooled data from three independent experiments (n = 6). ** p< 0.005; *** p < 0.0005 between the groups. **(F, G, H)** DCs from GL113 /1A8 treated *LdWT* and *LdCen^-/-^* infected mice ear dLN were flow sorted and were subjected to RNA isolation as described in Materials and Methods. Isolated total RNA was reverse transcribed and the expression of mRNA encoding cytokines (IL-12, TNF-a, IL-10) was evaluated by qPCR. The data were normalized to GAPDH expression. The data represent the mean values ± SD of results from 3 independent experiments that all yielded similar results (n = 5). ** p< 0.005; *** p < 0.0005 between the groups.

We next tested whether neutrophils are an important source of CCL3, affect DC recruitment, and its functional phenotype via depleting them using an anti-neutrophil mAb (1A8) as shown in the schematic diagram in Fig 5A. Isotype control antibody (GL113) treated mice served as a control for this experiment. Administration of 1A8 depleted ∼90% of Cd11b^+^Ly6G^+^ neutrophils in lymph nodes compared with GL113 treated groups on day 5 of infection (Fig 5B and 5C). Interestingly, with the depletion of neutrophils, there was a concomitant decrease in the CCL3 mRNA levels only in the lymph nodes from *LdCen*^-/-^ infected mice, and no significant reduction in lymph nodes from *LdWT* infected mice (Fig 5D). Next, we quantified the number of DCs in the ear dLNs of either PBS injected or *LdWT* or *LdCen^-/-^* infected mice treated with either 1A8 or GL113 antibodies. The gating strategy and individual flow plots are shown in S4A Fig. Depletion of neutrophils significantly decreased the number of DCs in the ear dLN of *LdCen^-/-^* infected mice whereas there was no decrease in the number of DCs in either *LdWT* infected mice or PBS injected mice (Fig 5E). These results demonstrate the essential role for neutrophils in the recruitment/migration of DCs during *LdCen^-/-^* immunization.

To further characterize the effect of neutrophil depletion on the phenotype of DCs, the parasitized DCs were sort selected from the ear dLNs of *LdWT* or *LdCen^-/-^* infected mice following GL113 or 1A8 treatment. The sorting strategy is identical as shown in S2J Fig. Uninfected DCs (Cd11b^+^Cd11c^+^Ly6G/Ly6C^-^MHCII^hi^) were sort selected from PBS injected mice. Neutrophil depletion significantly decreased the proinflammatory cytokine gene expression such as IL-12 (Fig 5F) and TNF-α (Fig 5G) along with a concomitant increase of IL-10 (Fig 5H) expression in DCs from *LdCen^-/-^* infected mice compared with GL113 treated mice. No significant alteration in cytokine expression was observed in *LdWT* infected mice under GL113 or 1A8 treated conditions (Fig 5F–5H).

**Fig 6:**
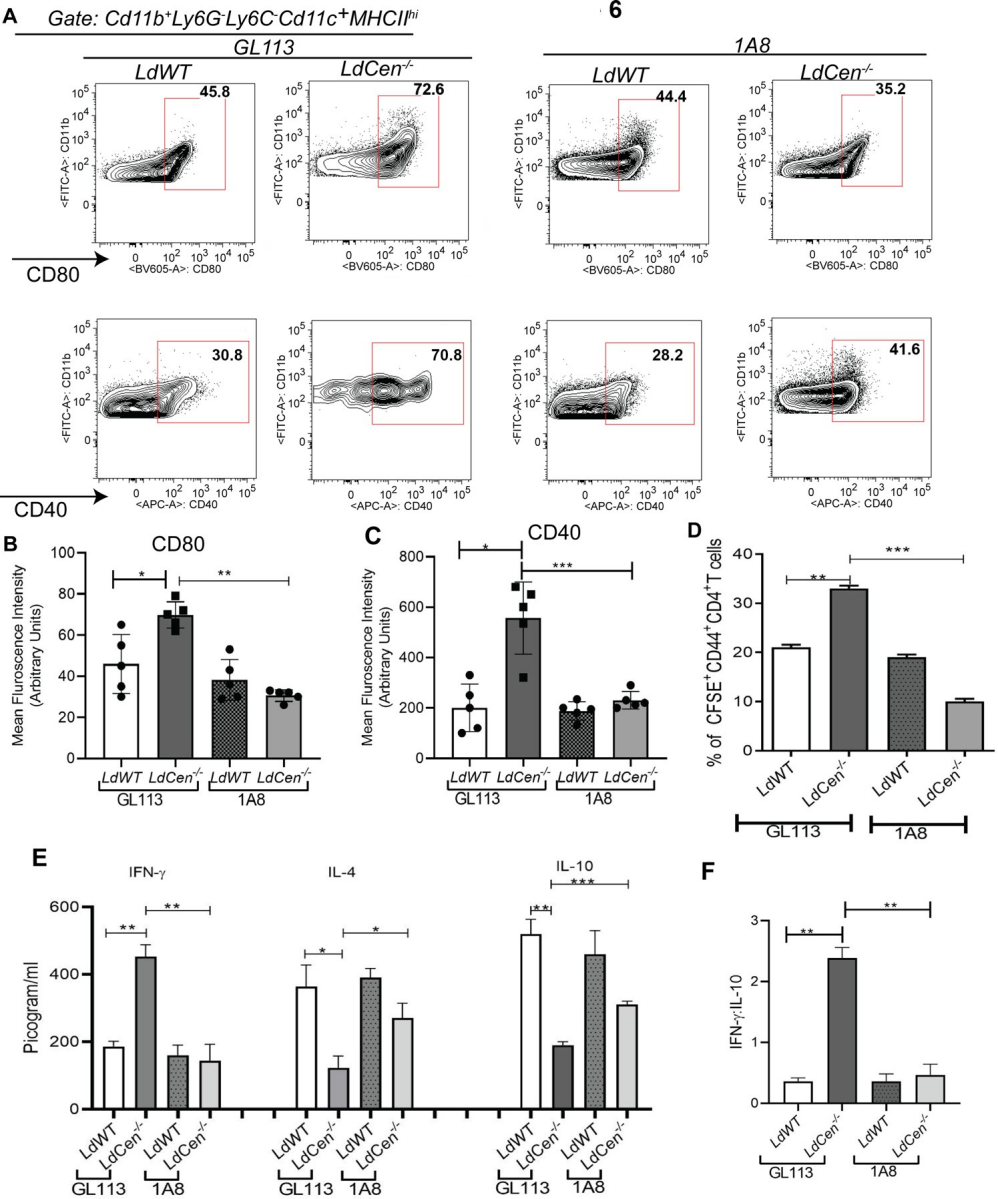
Neutrophil depletion abrogates DC mediated CD4^+^Th1 cell priming during *LdCen^-/-^* infection. **(A)** The expression of CD80 and CD40 in the DCs recruited in ear dLN of GL113/1A8 treated infected mice were analyzed by flow cytometry and represented by the flow plots. **(B, C)** The Mean fluorescence intensity **(**MFI) of the costimulatory molecules (CD80, CD40) expression in ear dLN has been represented by the bar diagram. The data represent the mean values ± SD of results from three independent experiments that all yielded similar results (n = 5). * p < 0.05; ** p < 0.005 *** p < 0.0005. **(D)** Parasitized DCs were flow sorted from the ear dLN of GL113/1A8 treated infected mice. Ag-specific CD4 T cell proliferation was estimated from DC-CD4 T cell coculture assay by studying CFSE dilution of gated CD4^+^CD44^+^T cells and is represented by the bar diagram. Cell proliferation was analyzed in triplicate experiments (n = 6). **(E)** The culture supernatant fluids were collected and assayed for IFN-γ (Analytical sensitivity: 15 pg/mL; Assay range:15–2,000 pg/mL) IL-4 (Analytical sensitivity: 4 pg/mL; Assay range: 4–500 pg/mL and IL-10 (Analytical sensitivity: 30 pg/mL, Assay range: 30–4,000 pg/mL) by ELISA. **(F)** IFN-γ/IL-10 ratio was determined. The data represent the picogram levels of cytokines in culture supernatants and are presented as means ± SD from three independent experiments that all yielded similar results (n = 6). * p < 0.05; ** p < 0.005 *** p < 0.0005.

Next, we analyzed the effect of neutrophil depletion on the functional activity of DCs. Cd11b^+^Cd11c^+^ Ly6G^-^ Ly6C^-^ MHCII^hi^ DCs were gated from the ear dLNs of PBS injected or *LdWT* or *LdCen^-/-^* infected mice treated with GL113/1A8 antibodies and the expression of activation markers such as (CD80, CD40) were measured. Corroborating our previous observation in Fig 2C, we could track the recruitment of similar number of total Cd11b^+^ cells in the ear dLNs of *LdWT/LdCen^-/-^* infected mice (S5A Fig). Neutrophil depletion significantly attenuated the expression of CD80 (Figs 6A, 6B and S5B) and CD40 (Figs 6A, 6C and S5C) (both MFI and % positivity) on DCs in *LdCen^-/-^* infected mice, whereas no significant reduction was observed in *LdWT* infected mice after neutrophil depletion (Figs 6A–6C and S5B and S5C). To further determine DCs antigen presentation to CD4^+^T cells, we sort selected uninfected DCs from PBS-injected mice or parasitized DCs from *LdWT^RFP^* or *LdCen^-/-^* ^mCherry^ infected mice recruited to the ear dLN (as shown in S2J Fig) under GL113 or 1A8 treated condition 5d post infection and were incubated with CFSE-labeled Ag-experienced CD4^+^ T cells for 5d. The gating strategy and individual flow plots have been shown in S5D Fig. The parasitized DCs from neutrophil depleted *LdCen^-/-^* infected mice showed a significant reduction in Ag specific CD4^+^T cell proliferation compared to the corresponding population from GL113 treated *LdCen^-/-^* infected mice, whereas no significant reduction was observed in *LdWT* infected mice with or without neutrophil depletion (Fig 6D).

We also measured cytokines from the supernatants of the DC-CD4^+^ T cell co-cultures. Parasitized DCs from 1A8 treated *LdCen^-/-^* infected mice upon coculture with CD4^+^ T cells showed significant attenuation of IFN-γ along with significant increase of IL-4 and IL-10 production compared to GL113 treated mice (Fig 6E). CD4^+^ T cells cocultured with DCs from 1A8 treated *LdCen^-/-^* infected mice displayed significantly lower IFN-γ/IL-10 ratios than CD4^+^ T cells cocultured with DCs from GL113 treated *LdCen^-/-^*infected mice (Fig 6F). In contrast, there was no effect in the cytokine levels between *LdWT* infected and neutrophil depleted animals (Fig 6E).

In order to further explore the role of neutrophils in DC mediated Th1 cell polarization *in vivo*, we also measured mRNA levels of T-bet, a lineage marker of Th1 cell differentiation [24], in CD4^+^ T cells from ear dLNs of GL113 or 1A8 treated *LdWT* and *LdCen^-/-^* infected mice 5d post infection (S6A Fig). T bet expression was significantly higher on CD4^+^ T cells in *LdCen^-/-^* infected mice compared with *LdWT* infected mice under GL113 treatment (S6B Fig). However, T bet expression was significantly attenuated in ear dLN derived CD4^+^ T cells after neutrophil depletion in *LdCen^-/-^*infected mice compared to non-depleted *LdCen^-/-^* infected mice (S6B Fig). Finally, such neutrophil-DC crosstalk in GL113 treated *LdCen^-/-^* infected mice resulted in significantly lower parasite burden in ear dLN compared to *LdWT* infected mice 7d post infection, an effect that was absent following neutrophil neutralization (S6C Fig).

Collectively, these data show that neutrophils play an important role in dictating DC mediated Th1 response by the *LdCen^-/-^* parasite immunization that could account for protective immunity by such parasites.

## Discussion

Recent studies provided novel insights into the mechanisms of neutrophil mediated activation of T cells [25]. These studies revealed that neutrophils may regulate T cell activation through direct effects [26–28] or via interacting with DCs, which may lead to DC activation, provide access to antigens captured by neutrophils, or proliferation and polarization of naïve T cells [29–31]. A previous study from our laboratory demonstrated the mechanisms of neutrophil mediated direct activation of CD4^+^ T cells during live attenuated *Leishmania* parasite infection [6]. However, neutrophil mediated immune regulation of DC functions and its subsequent effect on T cell activation during live attenuated *Leishmania* parasite infection remains unexplored. In this study we report that while *LdWT* parasite infected neutrophils attenuate DC activation, *LdCen^-/-^* infected neutrophils result in heightened DC activation and effective Ag presentation to CD4^+^ T cells. The crosstalk between neutrophil and DC upstream of T lymphocytes is strikingly different between *LdWT* or *LdCen^-/-^* parasite infections and thus plays an important role in shaping *LdCen^-/-^* induced protective immunity.

The key role of neutrophils in DC recruitment through the synthesis of chemokines has been documented in several parasitic and bacterial infections [32]. For example, exposure to the protozoan parasite *Toxoplasma gondii*, triggers the neutrophils to release DC-attracting chemokines CCL3, CCL4, CCL5, and CCL20 [7]. Likewise, *Mycobacteria* are reported to induce the release of DC-attracting chemokines by neutrophils, and depletion of neutrophils in infected mice delayed DC migration to the dLNs [10]. Notably, following *L*. *major* inoculation, neutrophil derived CCL3 is shown to be crucial in the early recruitment of DCs to the dermis that will further direct the development of an adaptive immune response to *L*. *major*. Furthermore, *L*. *major*-infected CCL3^-/-^ mice, adoptively reconstituted with WT neutrophils, were able to chemoattract DCs to the site of parasite inoculation, demonstrating that neutrophil-derived CCL3 contributes to the early DC recruitment to the inoculation site [8]. In our study, we first identified neutrophil derived CCL3 as an essential chemoattractant for DCs during *L*. *donovani* infection. Importantly, following *LdCen^-/-^* infection, neutrophils showed a much higher level of induction of CCL3 *in vitro* and subsequently attracted a larger number of DCs compared to *LdWT* infection. Further, inhibition of CCL3 in *LdCen^-/-^* infected neutrophil impeded DC migration. Thus, the activation of CCL3 in *LdCen^-/-^* infected neutrophil is associated with heightened DC migration. Next, we considered whether the interaction between DCs and neutrophils, especially infected neutrophils, might modulate DC function. DC migration to the lymph nodes and maturation are crucial for antigen delivery and activation of T cell responses [33]. We observed *LdWT* parasite infected neutrophils are proficient at immune subversion and thus boosting of CCL3 alone may not be adequate to enable the activation of dendritic cells to clear the *LdWT* parasites acquired upon engulfment of neutrophils. However, as opposed to *LdWT* parasites, *LdCen^-/-^* parasites might lack the virulence factor(s) and thus fail to suppress DC activation. Indeed, we observed that *LdCen^-/-^* infected neutrophils induced enhanced DC activation as indicated by elevated expression of costimulatory molecules concomitant with an induction of pro-inflammatory IL-12 secretion which may result in the increased capacity of these DCs to induce Th1 cell proliferation.

The *in vitro* results described above were further validated by *in vivo* experiments in an intradermal ear model of infection. Consistent with our *in vitro* results, we also observed that neutrophils responding to *LdCen^−/−^* parasites in ear dLN augmented CCL3 expression, which played a critical role in attracting DCs 5d post infection. Interestingly, in *LdCen^-/-^* infected mice neutrophil derived CCL3 also has a predominant role in the migration of Cd11b^+^MHCII^hi^CD11c^+^CD8α^−^CD205^low^DC, the major DC population that primes CD4^+^ T cell response in dLNs previously described in *L major* studies [34]. However, it must be noted that protective immunity induced by *LdCen^-/-^* immunization has been shown to be mediated by both CD4^+^ and CD8^+^ T cells [35] implying the importance of Cd11b^+^CD11c^+^Ly6G/Ly6c^-^ MHCII^hi^DC population in the vaccine context. Although, same number of total myeloid /Cd11b^+^ cells migrated to the ear dLN in both groups of infected mice, neutrophils from *LdCen^-/-^* infected mice were potent in achieving a heightened DC migration in dLN due to elevated CCL3 production. It is worth mentioning here, besides these subtypes of DCs studied by us, recent study highlights monocyte-derived dendritic cell-like phagocyte subset (Ly6C^+^CCR2^+^ monocytes with high CD11c expression) serve as a predominant reservoir for efficient intracellular multiplication of *L*. *major* and spread to the new host cells [36]. However, the interaction of neutrophils with these subtypes of DCs and their subsequent role in *Leishmania* vaccine immunity warrants further investigation.

Notably, we also observed that neutrophils responding to *LdCen^−/−^* parasites in the ear (the site of parasite inoculation) augmented the expression of CCL3 which attracted significantly higher number of dermal DCs 24 h post infection. However, DCs are professional APCs playing a key role in launching and regulation of the immune response via interacting with T cells that occurs in lymph nodes (LNs) [37]. Hence, we focused on whether crosstalk between neutrophils and DCs in the ear draining LN may have a direct impact on the development of *LdCen*^-/-^ specific immune response and thereby, on the out-come of infection.

During pathogenic infection, neutrophils and DCs, normally positioned in distinct anatomical compartments, may colocalize at sites of inflammation. Neutrophils are short-lived cells that must be targeted for orderly removal upon apoptotic death. The function of DCs in the clearance of dying neutrophils is central to the resolution of infection that will in most instances affect antigen-presenting properties of DCs [11]. Generally, recognition and engulfment of apoptotic neutrophils by DCs is known to inhibit their production of pro-inflammatory cytokines, expression of costimulatory molecules, and their ability to stimulate T-cell proliferation [38–40]. Notably, majority of the infected DCs recovered from the skin immediately after *L*. *major* infection acquire their infections via capture of infected neutrophils, and the sequestration of *L*. *major* antigens within apoptotic neutrophils would seem an especially efficient process for inhibiting DC activation and their ability to initiate the *Leishmania-*specific T-cell response [9]. In contrast, there are observations which demonstrate the mechanisms of neutrophils in promoting adaptive immunity by delivering antigens to DCs and making DCs more effective initiators of naive CD4^+^ T cell activation [10,41]. For example, in the context of mycobacterial antigens, it has been observed that neutrophils promote adaptive immune responses to *M*. *tuberculosis* by delivering *M*. *tuberculosis* to DCs in a form that makes DCs more effective initiators of naive CD4 T cell activation [10]. In our study, we demonstrated the uptake of *LdWT* or *LdCen^-/-^* infected neutrophils by DCs in the ear dLN 5d post infection of LYS-eGFP mice. The majority of the Cd11c^+^DCs (termed P1 DCs) recovered from the ear dLN at 5d post infection were positive for both the RFP and eGFP signals and stained positive for neutrophil markers. This demonstrated that the majority of the infected DCs acquired their parasites via engulfment of infected neutrophils similar to studies in *M*. *tuberculosis* [10]. Notably, DCs that ingested *LdWT/LdCen^-/-^* parasite infected neutrophils were better activated than the DCs (termed P2 DCs) that acquire the parasites independent of neutrophils. Interestingly, DCs that ingested *LdCen^-/-^* infected neutrophils in ear dLN 5d post infection were higher in numbers and showed significant induction of costimulatory molecules and proinflammatory cytokines compared to DCs bearing *LdWT* infected neutrophils. We further addressed the consequences of *LdWT/ LdCen^-/-^* infected neutrophil capture by DCs on the latter’s ability to present parasite-derived antigen to CD4^+^ T cells *ex-vivo*. Corroborating with our *in vitro* observation, unlike *LdWT* parasites, *LdCen^-/-^* parasite infected neutrophils were defective in their capacity to attenuate the activation of DC. Thus, significant enhancement of DC mediated CD4^+^T cell activation following capture of *LdCen^-/-^* infected neutrophil occurred compared to DCs that ingested *LdWT* infected neutrophils. In either case it was significantly higher than respective DCs that acquire the parasites directly. Consequently, our data draw a clear distinction between the ability of *LdCen^-/-^* infected and *LdWT* infected neutrophils to deliver activation signals to DCs in the ear dLN

Further, the uptake of *LdCen^-/-^* containing neutrophils was essential for *LdCen^-/-^* to enhance DC maturation and to initiate the anti-*Leishmania* CD4^+^T cell response. The less potent immune response observed in P2 DCs that directly acquired either *LdWT* or *LdCen*^-/-^ parasites highlights the novel findings from our study. Further, our data shows that neutrophils may not simply be an efficient mechanism of parasite delivery as illustrated in other pathogens but that their immunoregulatory role in enhancing the DCs activation towards APC activity is critical in producing potent anti-parasitic mechanisms induced by *LdCen*^-/-^ infection.

To summarize, the importance of neutrophils-DC interaction in *LdCen^-/-^* parasite mediated immunity was further validated through the observation that depletion of neutrophils results in decreased recruitment of DCs to dLNs. This finding is in accordance with the study by Charmoy et al. 2010 indicating that neutrophils are essential for DC recruitment to the ear dermis following *L*. *major* inoculation [8]. It is important to note that as opposed to 1A8 antibody which selectively targets mouse neutrophils [9], depletion of neutrophils using NIMP-R14 can have significant off-target outcomes, including 1) depletion of non-neutrophils (especially monocytes, the source of recruited DCs); 2) neutrophil ‘rebound’ once the depleting antibody is cleared; and 3) compensatory recruitment of other inflammatory cells. It is important to note that using NIMP-R14 antibodies may affect other cells that may have a role in immune response. We also observed, neutrophil depletion delays activation/ maturation of DCs in ear dLN of *LdCen^-/-^* infected mice as indicated by the presence of lower percentages of activated DCs (CD80^+^ and CD40^+^) along with attenuated expression of cytokines such as IL-6, TNF and heightened expression of IL-10 which subsequently compromised the proliferation of naive Ag-specific CD4^+^ Th1 cells *ex-vivo*. The consequence of this inhibition in *Leishmania* specific host protective CD4^+^ Th1 cell priming was directly supported by the attenuated Tbet expression in ear dLN derived CD4^+^ T cells in neutrophil depleted mice consistent with the studies in *M*. *tuberculosis* infection where depletion of neutrophils resulted in decreased migration of DCs to dLNs and delayed activation and proliferation of naive Ag-specific CD4^+^ T cells in dLNs of mice [10]. We currently do not know the mechanism of neutrophil mediated expression of activation markers by DCs in dLN in *LdCen^-/-^* infected mice. However, it has been reported earlier that neutrophils expressing CCR7 can migrate to the lymph nodes via the lymphatics rendering activation/maturation of DCs via upregulation of MHC-II, CD40, CD80 and CD86 molecules as well as IL-12, TNF-α and IL-6 secretion [25]. Previously we have reported significantly higher CCR7 expression in *LdCen^-/-^* infected neutrophils compared to *LdWT* infected neutrophils [6]. It is possible that following *LdCen^-/-^* infection significantly higher number of neutrophils migrated to the lymph nodes and resulted in higher DC activation/maturation compared to *LdWT* as indicated by significant upregulation of maturation marker/costimulatory molecules which was abrogated in neutrophil depleted mice. Further, significantly higher DC numbers in ear dLN of *LdCen^-/-^* infected mice indicate that neutrophil derived CCL3 generation facilitated increased DC migration and subsequent recruitment to the dLN of these mice. Notwithstanding, we will be pursuing this detailed line of research in the future. Collectively, neutrophils in *LdCen^-/-^* infected mice facilitate enhanced DC migration and subsequent recruitment along with DC activation/maturation in dLN and confers them with the capacity for efficient priming of Th1 cells.

The role of neutrophils in providing direct stimulatory cues to effector CD4^+^ Th1 cells involved in protection by live attenuated *Leishmania* vaccine against virulent *L*. *donovani* challenge has been reported recently by our group [6]. Additionally, the current findings demonstrate that neutrophils also predominately regulate CD4^+^ T cell immune response by affecting the function of DCs during *LdCen^-/-^* infection and such cross talk involving neutrophils and DCs results in significantly lower *LdCen^-/-^* parasite numbers at early time point via enhanced effector response and might cumulatively contribute towards generating live attenuated *Leishmania* vaccine–induced protective immunity as observed earlier [6]. Conversely, *LdWT* parasites utilizes such neutrophil-DC crosstalk to subvert the host effector response which enables parasite proliferation in the host.

In summary, our results indicate that *LdWT* and *LdCen^-/-^* parasites deploy differential immune strategies to modulate DC function. While *LdWT* parasite infected neutrophils cause diminution of DC activation, *LdCen^-/-^* parasite infected neutrophils interact with DC, generates activation of adaptive immune response. Thus, the impact of the early neutrophil- DC interactions described in this study clearly highlights the immunological differences exist between a virulent and live attenuated parasite in modulation of DC response and may be especially relevant in future vaccine development against Leishmaniasis.

## Supporting information

S1 Fig No significant differences in the parasite numbers and CCL4 and CCL5 mRNA expression between *LdWT* and *LdCen^-/-^* infections of neutrophils *in vitro*. (**A)** qRT-PCR assay showing the parasite burden in the neutrophils infected with *LdWT* or *LdCen^−/−^* parasites for 16h. (**B)** Peritoneal neutrophils were infected with *LdWT/LdCen^-/-^* parasites for 16h. Intracellular parasite numbers were visualized by Giemsa staining and estimated microscopically. The infection efficiency (percentage of infected cells) was recorded. (**C**) qRT-PCR assay showing the parasite burden in the neutrophils infected with *LdWT* or *LdCen^−/−^* parasites for 24h. (**D**) Peritoneal neutrophils were infected with *LdWT/LdCen^-/-^* parasites for 24h. Intracellular parasite numbers were visualized by Giemsa staining and estimated microscopically. The infection efficiency (percentage of infected cells) was recorded. To measure parasite load in these cultures, a minimum of 300 neutrophils were counted. The data represent the mean values ± SD of results from three independent experiments that all yielded similar results. **(E, F)** Peritoneal neutrophils were either left uninfected or infected with *LdWT/LdCen^-/-^* parasites for 16h. Changes in the mRNA expression levels of CCL4 and CCL5 from uninfected and infected neutrophils were determined by qPCR as described in Materials and Methods. The data represent the mean values ± SD of results from three independent experiments that all yielded similar results. *p< 0.05; ** p < 0.005. **(G)** BMDCs were cocultured with LPS activated uninfected or infected neutrophils as described in Materials and Methods. The percentages of MHCII and CD40 positive DCs were reported. The data represent the mean values ± SD of results from three independent experiments that all yielded similar results. *p< 0.05; ** p < 0.005 *** p < 0.0005.
(PDF)

S2 Fig Sorting strategy showing the sort selection of parasitized neutrophils and DCs. **No significant differences in the CCL4 and CCL5 mRNA expression was observed in sort selected parasitized neutrophils from ear dLN 5d post infection with *LdWT* or *LdCen-/-*. (A)** The FMO’s for all the fluorophores used in experiment (B&J) have been shown and are based on the same gating as in (B&J). **(B)** Sorting strategy showing parasitized neutrophils isolation from ear dLN. **(C)** Post sort analysis for the sort selected parasitized neutrophils has been shown. **(D)** Confocal microscopic image of the sort selected parasitized neutrophils stained with Hoechst nuclear dye with excitation 561 and 405 nm for red and blue channel have been shown. Scale bar 1μm. **(E)** Confocal microscopic image of the sort selected parasitized neutrophils with excitation 670, 590 and 405 nm for alexa700, mRFP and Pacific blue channels imaging respectively have been shown. Scale bar 2μm. **(F, G)** mRNA expression levels of CCL4 and CCL5 from sorted neutrophils in the ear dLN were estimated 5d post infection by qPCR and expressed as fold change from uninfected naive mice (n = 5). The experiment was repeated three times with pooled digests from eight to twelve ear dLNs per experiment. The data represent the mean values ± SD of results from three independent experiments that all yielded similar results. **(H)** The gating strategy and **(I)** changes in the total number of DCs (CD11b^+^CD11c^+^MHCII^hi^CD205^low^CD8α^-^) per ear dLN have been shown under CCL3 non-depleted or depleted condition 5d post infection. Values shown are the mean numbers of cells per ear dLN ± SD of results. 6–8 ear dLN, pooled data from 3 independent experiments that all yielded similar results (n = 6). *p< 0.05; ** p < 0.005 *** p < 0.0005. **(J)** Parasitized DCs were flow sorted from the ear dLN 5d post infection. The sorting strategy has been displayed. **(K)** Post sort analysis for the sort selected parasitized DCs has been shown. **(L)** Confocal microscopic image of the sort selected parasitized DCs have been shown. Scale bar 2μm.
(PDF)

S3 Fig Negligible T cell proliferation was observed in infected P2-DC and CD4T cell coculture set. **(A)** Gating strategy for flow cytometry analysis and representative flow plots showing the expression of CD40 in P1 and P2 DCs in ear dLN of LYS-eGFP infected mice. **(B)** Ag-specific CD4 T cell proliferation was estimated from P2 DC–CD4 T cell coculture assay by studying CFSE dilution of gated CD4^+^CD44^+^ T cells and is represented by the histogram.
(PDF)

S4 Fig Kinetics of DC recruitment in GL113 or 1A8 treated mice following infection with *LdWT/LdCen^-/-^*. **(A)** The gating strategy and individual flow plots for DCs (Cd11b^+^Cd11c^+^Ly6G^-^Ly6C^-^MHCII^hi^) recruitment in ear dLN has shown at 5-day post infection in mice depleted or not of neutrophils.
(PDF)

S5 Fig DCs from neutrophil depleted *LdCen^-/-^* infected mice exhibited compromised CD4T cell priming ability *ex-vivo*. **(A)** Total number of Cd11b^+^ cells recruited in ear dLN 5 days post infection in either GL113 or 1A8 treated mice has been shown. The experiment was repeated three times with pooled digests from five to six ear dLNs per experiment. The data represent the mean values ± SD of results from three independent experiments that all yielded similar results. **(B, C)** The percentages of CD80 and CD40 positive DCs in ear dLN of GL113/1A8 treated *LdWT/LdCen^-/-^* infected mice 5d post infection was reported. **(D)** Gating strategy showing CFSE dilution of gated CD4^+^CD44^+^T cells from DC-CD4 T cell coculture assay.
(PDF)

S6 Fig Neutrophil depleted immunized mice ear dLN derived CD4 T cells exhibit attenuated Tbet expression and exacerbated parasite numbers. **(A)** Schematic diagram showing experimental scheme for neutrophil depletion and subsequent experiments. (**B**) Real-time PCR analysis of RNA isolated from purified CD4 LN T lymphocytes at 5d post infection as described in Materials and Methods is shown. Normalized expression levels of Tbet were estimated. Data are presented as fold change from uninfected naive mice. The data represent the mean values ± standard deviations of results from 3 independent experiments that all yielded similar results. *, P < 0.05; **, P < 0.005. (n = 6). **(C)** Parasite numbers in ear dLNs of different groups of GL113/1A8 treated infected mice were measured 7 days post infection. Means and standard errors of the means for 5 mice in each group are shown. Data are representative of two independent experiments. *, P < 0.05.
(PDF)
