# Supplementary material for: Neutrophil-dendritic cell interaction plays an important role in live attenuated Leishmania vaccine induced immunity
Source: PLoS Negl Trop Dis. 2022 Feb 22;16(2):e0010224. doi: 10.1371/journal.pntd.0010224 (PMC8896671; doi:10.1371/journal.pntd.0010224)
Supplement: S1 Fig — (A) qRT-PCR assay showing the parasite burden in the neutrophils infected with LdWT or LdCen−/− parasites for 16h. (B) Peritoneal neutrophils were infected with LdWT/LdCen-/- parasites for 16h. Intracellular parasite numbers were visualized by Giemsa staining and estimated microscopically. The infection efficiency (percentage of infected cells) was recorded. (C) qRT-PCR assay showing the parasite burden in the neutrophils infected with LdWT or LdCen−/− parasites for 24h. (D) Peritoneal neutrophils were infected with LdWT/LdCen-/- parasites for 24h. Intracellular parasite numbers were visualized by Giemsa staining and estimated microscopically. The infection efficiency (percentage of infected cells) was recorded. To measure parasite load in these cultures, a minimum of 300 neutrophils were counted. The data represent the mean values ± SD of results from three independent experiments that all yielded similar results. (E, F) Peritoneal neutrophils were either left uninfected or infected with LdWT/LdCen-/- parasites for 16h. Changes in the mRNA expression levels of CCL4 and CCL5 from uninfected and infected neutrophils were determined by qPCR as described in Materials and Methods. The data represent the mean values ± SD of results from three independent experiments that all yielded similar results. *p< 0.05; ** p < 0.005. (G) BMDCs were cocultured with LPS activated uninfected or infected neutrophils as described in Materials and Methods. The percentages of MHCII and CD40 positive DCs were reported. The data represent the mean values ± SD of results from three independent experiments that all yielded similar results. *p< 0.05; ** p < 0.005 *** p < 0.0005. (PDF) [file pntd.0010224.s001.pdf]

# *S*1 Fig

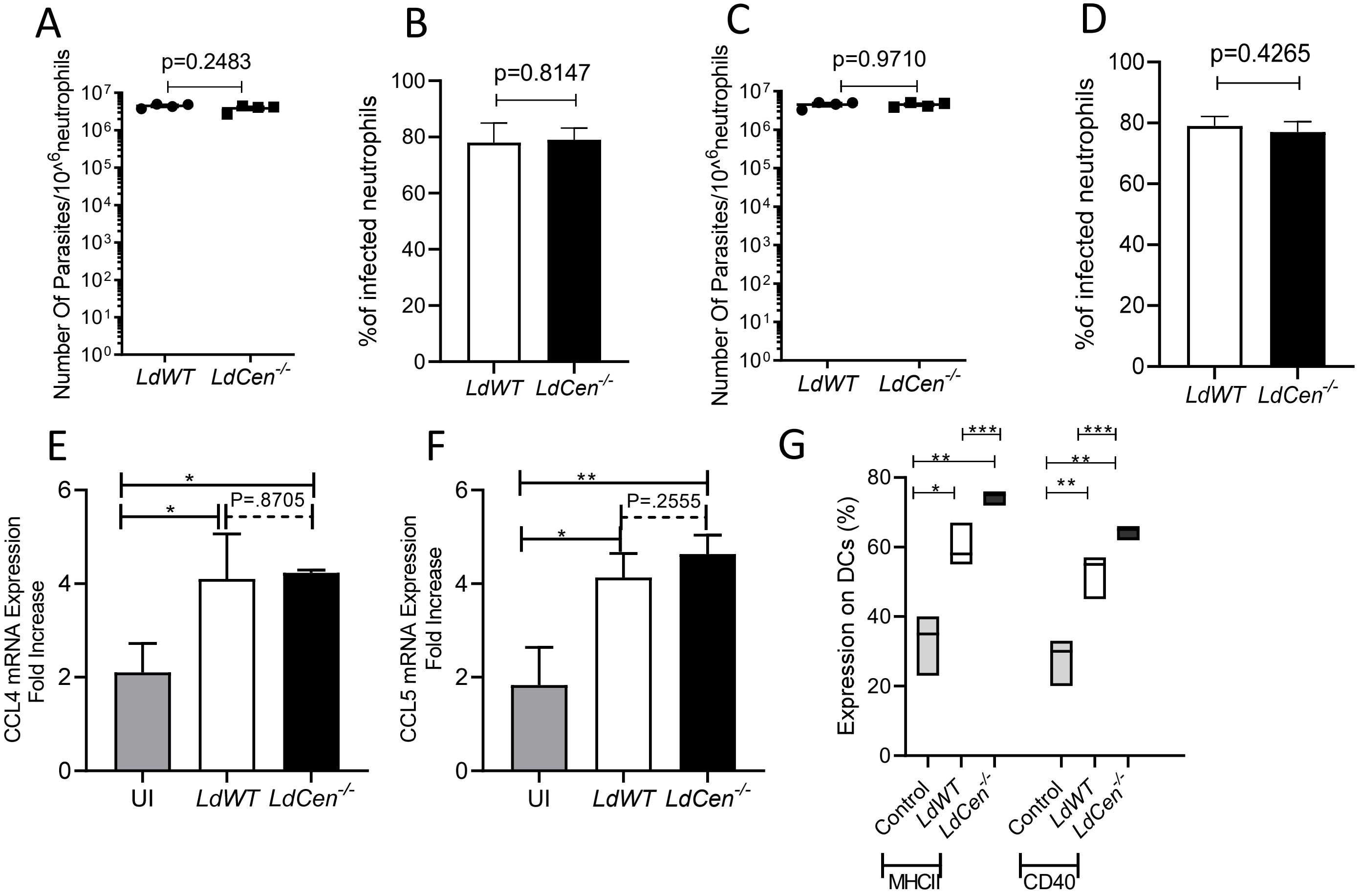

A
p=0.2483
Number Of Parasites/10^6neutrophils
LdWT
LdCen-/-
B
p=0.8147
%of infected neutrophils
C
p=0.9710
D
p=0.4265
E
CCL4 mRNA Expression
Fold Increase
P=.8705
UI
F
CCL5 mRNA Expression
P=.2555
G
Expression on DCs (%)
Control
MHCII
CD40

***Supporting Information S1: No significant differences in the parasite numbers and CCL4 and CCL5 mRNA expression between LdWT and LdCen$^{-/-}$ infections of neutrophils in vitro.*** *(**A**) qRT-PCR assay showing the parasite burden in the neutrophils infected with LdWT or LdCen$^{-/-}$ parasites for 16h. (**B**) Peritoneal neutrophils were infected with LdWT/LdCen$^{-/-}$ parasites for 16h. Intracellular parasite numbers were visualized by Giemsa staining and estimated microscopically. The infection efficiency (percentage of infected cells) was recorded. (**C**) qRT-PCR assay showing the parasite burden in the neutrophils infected with LdWT or LdCen$^{-/-}$ parasites for 24h. (**D**) Peritoneal neutrophils were infected with LdWT/LdCen$^{-/-}$ parasites for 24h. Intracellular parasite numbers were visualized by Giemsa staining and estimated microscopically. The infection efficiency (percentage of infected cells) was recorded. To measure parasite load in these cultures, a minimum of 300 neutrophils were counted. The data represent the mean values ± SD of results from three independent experiments that all yielded similar results. **(E,F)** Peritoneal neutrophils were either left uninfected or infected with LdWT/LdCen$^{-/-}$ parasites for 16h. Changes in the mRNA expression levels of CCL4 and CCL5 from uninfected and infected neutrophils were determined by qPCR as described in Materials and Methods. The data represent the mean values ± SD of results from three independent experiments that all yielded similar results. $*p< 0.05$; $** p < 0.005$ **(G)** BMDCs were cocultured with LPS activated uninfected or infected neutrophils as described in Materials and Methods. The percentages of MHCII and CD40 positive DCs were reported. The data represent the mean values ± SD of results from three independent experiments that all yielded similar results. $*p< 0.05$; $** p < 0.005$ $*** p < 0.0005$*
