# Supplementary material for: Neutrophil-dendritic cell interaction plays an important role in live attenuated Leishmania vaccine induced immunity
Source: PLoS Negl Trop Dis. 2022 Feb 22;16(2):e0010224. doi: 10.1371/journal.pntd.0010224 (PMC8896671; doi:10.1371/journal.pntd.0010224)
Supplement: S2 Fig — No significant differences in the CCL4 and CCL5 mRNA expression was observed in sort selected parasitized neutrophils from ear dLN 5d post infection with LdWT or LdCen-/-. (A) The FMO’s for all the fluorophores used in experiment (B&J) have been shown and are based on the same gating as in (B&J). (B) Sorting strategy showing parasitized neutrophils isolation from ear dLN. (C) Post sort analysis for the sort selected parasitized neutrophils has been shown. (D) Confocal microscopic image of the sort selected parasitized neutrophils stained with Hoechst nuclear dye with excitation 561 and 405 nm for red and blue channel have been shown. Scale bar 1μm. (E) Confocal microscopic image of the sort selected parasitized neutrophils with excitation 670, 590 and 405 nm for alexa700, mRFP and Pacific blue channels imaging respectively have been shown. Scale bar 2μm. (F, G) mRNA expression levels of CCL4 and CCL5 from sorted neutrophils in the ear dLN were estimated 5d post infection by qPCR and expressed as fold change from uninfected naive mice (n = 5). The experiment was repeated three times with pooled digests from eight to twelve ear dLNs per experiment. The data represent the mean values ± SD of results from three independent experiments that all yielded similar results. (H) The gating strategy and (I) changes in the total number of DCs (CD11b+CD11c+MHCIIhiCD205lowCD8α-) per ear dLN have been shown under CCL3 non-depleted or depleted condition 5d post infection. Values shown are the mean numbers of cells per ear dLN ± SD of results. 6–8 ear dLN, pooled data from 3 independent experiments that all yielded similar results (n = 6). *p< 0.05; ** p < 0.005 *** p < 0.0005. (J) Parasitized DCs were flow sorted from the ear dLN 5d post infection. The sorting strategy has been displayed. (K) Post sort analysis for the sort selected parasitized DCs has been shown. (L) Confocal microscopic image of the sort selected parasitized DCs have been shown. Scale bar 2μm. (PDF) [file pntd.0010224.s002.pdf]

S2 Fig

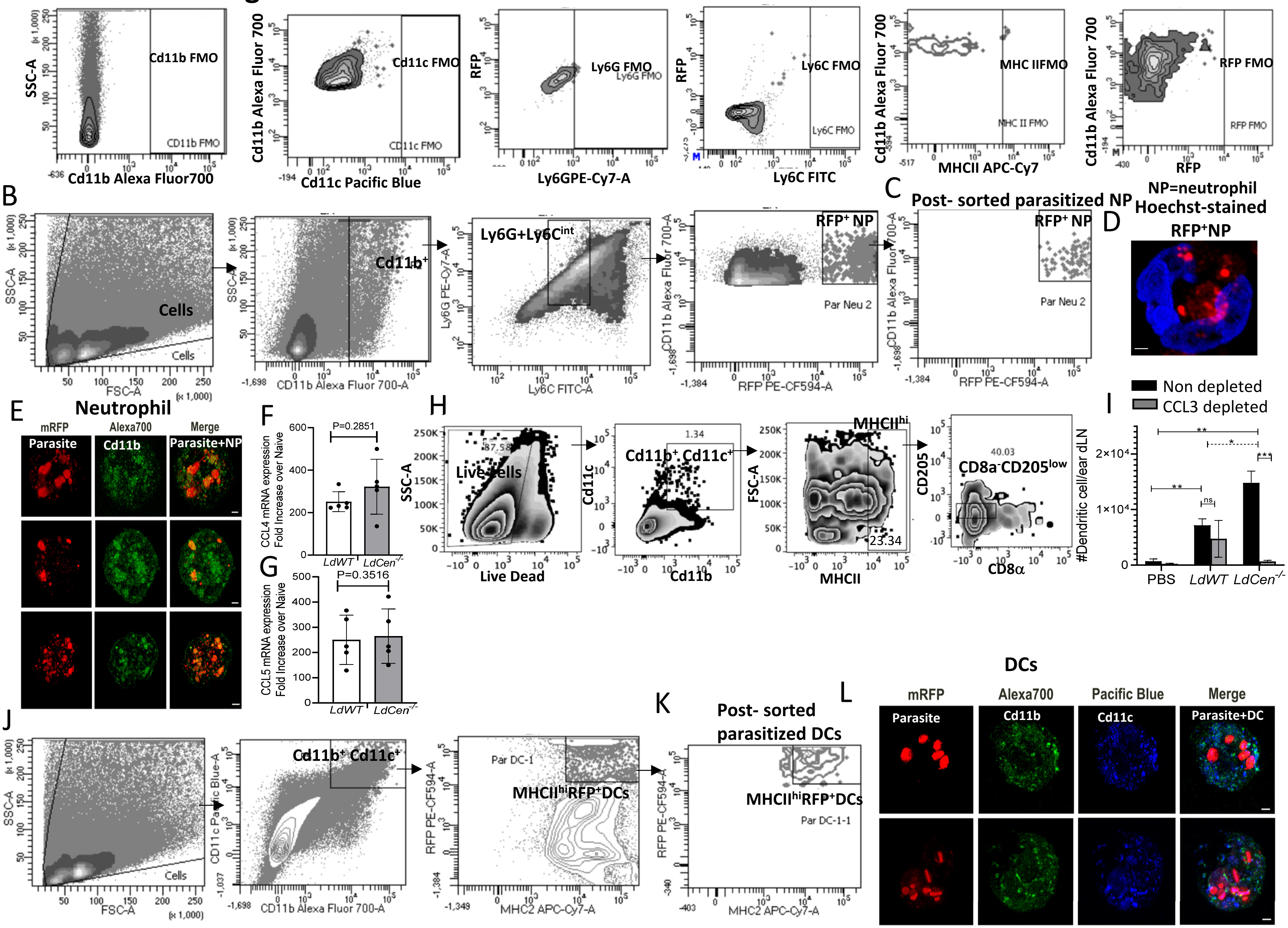

A
Cd11b FMO
SSC-A
Cd11b Alexa Fluor700
Cd11c FMO
Cd11b Alexa Fluor 700
Cd11c Pacific Blue
Ly6G FMO
RFP
Ly6GPE-Cy7-A
Ly6C FMO
Ly6C FITC
MHC IIFMO
MHCII APC-Cy7
RFP FMO
Cd11b Alexa Flour 700
RFP
B
Cells
Cd11b+
Ly6G+Ly6Cint
RFP+ NP
Par Neu 2
FSC-A
CD11b Alexa Fluor 700-A
Ly6C FITC-A
RFP PE-CF594-A
C
Post- sorted parasitized NP
RFP+ NP
NP=neutrophil
D
Hoechst-stained
RFP+NP
E
Neutrophil
mRFP
Alexa700
Merge
Parasite
Cd11b
Parasite+NP
F
CCL4 mRNA expression Fold Increase over Naive
P=0.2851
LdWT
LdCen-/-
G
CCL5 mRNA expression Fold Increase over Naive
P=0.3516
H
Live cells
Live Dead
Cd11b+ Cd11c+
Cd11b
Cd11c
MHCIIhi
MHCII
FSC-A
CD8a-CD205low
CD8α
CD205
I
Non depleted
CCL3 depleted
#Dendritic cell/ear dLN
PBS
LdWT
LdCen-/-
J
Cd11b+ Cd11c+
MHCIIhiRFP+DCs
CD11c Pacific Blue-A
CD11b Alexa Fluor 700-A
RFP PE-CF594-A
MHC2 APC-Cy7-A
K
Post- sorted parasitized DCs
MHCIIhiRFP+DCs
L
DCs
mRFP
Alexa700
Pacific Blue
Merge
Parasite
Cd11b
Cd11c
Parasite+DC

Supporting Information S2: Sorting strategy showing the sort selection of parasitized neutrophils and DCs. No significant differences in the CCL4 and CCL5 mRNA expression was observed in sort selected parasitized neutrophils from ear dLN 5d post infection with LdWT or LdCen$^{-/-}$. ***(A)*** *The FMO's for all the fluorophores used in experiment (B&I) have been shown and are based on the same gating as in (B&I).* ***(B)*** *Sorting strategy showing parasitized neutrophils isolation from ear dLN.* ***(C)*** *Post sort analysis for the sort selected parasitized neutrophils has been shown.* ***(D)*** *Confocal microscopic image of the sort selected parasitized neutrophils stained with Hoechst nuclear dye with excitation* 561 and 405 nm for red and blue channel *have been shown. Scale bar 1μm.* ***(E)*** *Confocal microscopic image of the sort selected parasitized neutrophils with excitation 670, 590 and 405 nm for alexa700, mRFP and Pacific blue channels imaging respectively have been shown. Scale bar 2μm.* ***(F,G)*** *mRNA expression levels of CCL4 and CCL5 from sorted neutrophils in the ear dLN were estimated 5d post infection by qPCR and expressed as fold change from uninfected naive mice (n = 5). The experiment was repeated three times with pooled digests from eight to twelve ear dLNs per experiment. The data represent the mean values ± SD of results from three independent experiments that all yielded similar results.* ***(H)*** *The gating strategy and* ***(I)*** *changes in the total number of DCs ($CD11b^+$ $CD11c^+MHCII^{hi}CD205^{low}CD8a^-$)per ear dLN have been shown under CCL3 non-depleted or depleted condition 5d post infection. Values shown are the mean numbers of cells per ear dLN ± SD of results. 6–8 ear dLN, pooled data from 3 independent experiments that all yielded similar results (n=6).*$p< 0.05$; ** $p < 0.005$ *** $p < 0.0005$.* ***(J)*** *Parasitized DCs were flow sorted from the ear dLN 5d post infection. The sorting strategy has been displayed.* ***(K)*** *Post sort analysis for the sort selected parasitized DCs has been shown.* ***(L)*** *Confocal microscopic image of the sort selected parasitized DCs have been shown. Scale bar 2μm.*
