# Supplementary material for: Neutrophil-dendritic cell interaction plays an important role in live attenuated Leishmania vaccine induced immunity
Source: PLoS Negl Trop Dis. 2022 Feb 22;16(2):e0010224. doi: 10.1371/journal.pntd.0010224 (PMC8896671; doi:10.1371/journal.pntd.0010224)
Supplement: S3 Fig — (A) Gating strategy for flow cytometry analysis and representative flow plots showing the expression of CD40 in P1 and P2 DCs in ear dLN of LYS-eGFP infected mice. (B) Ag-specific CD4 T cell proliferation was estimated from P2 DC–CD4 T cell coculture assay by studying CFSE dilution of gated CD4+CD44+ T cells and is represented by the histogram. (PDF) [file pntd.0010224.s003.pdf]

## *S*3 Fig

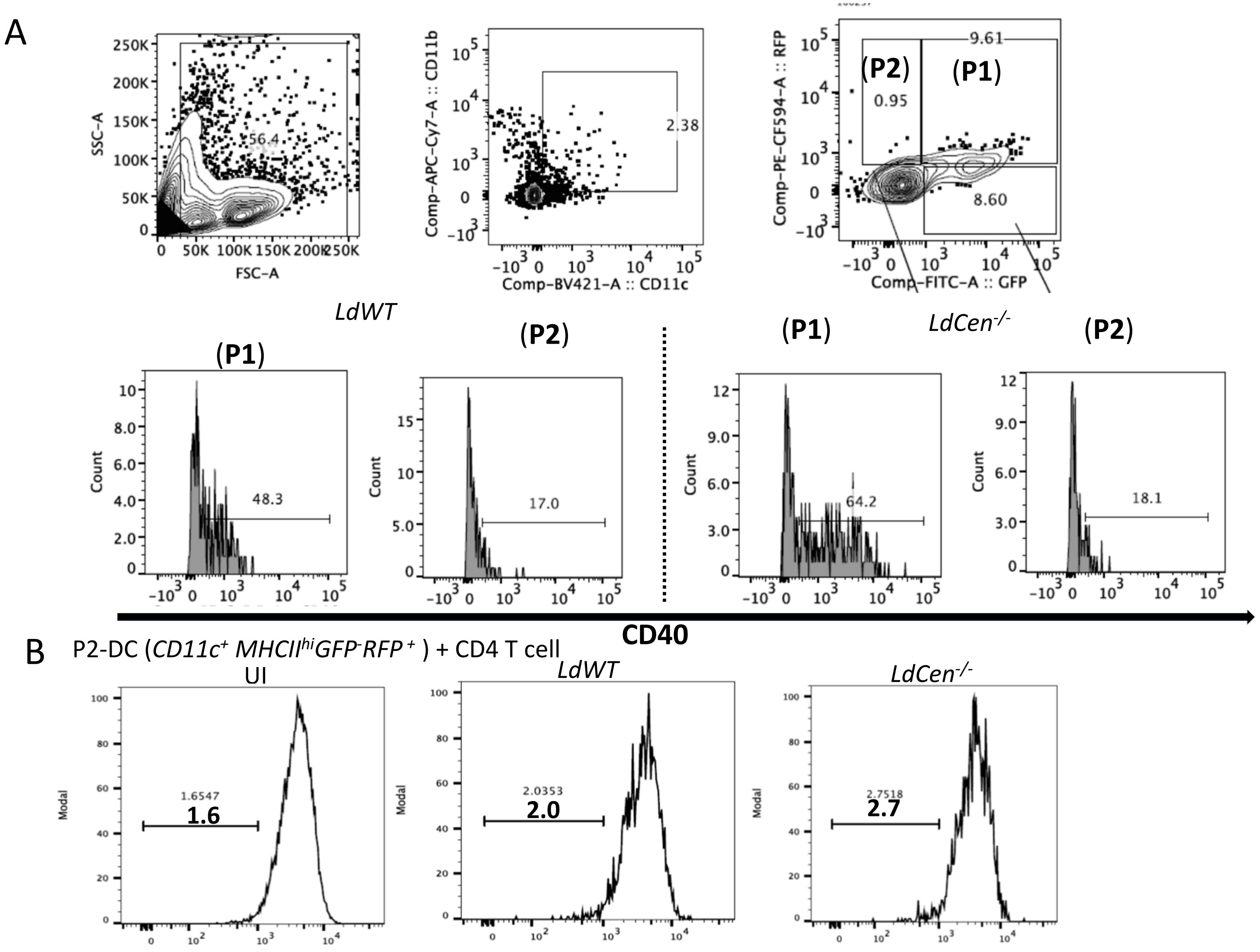
A
SSC-A
FSC-A
56.4
Comp-APC-Cy7-A :: CD11b
Comp-BV421-A :: CD11c
2.38
Comp-PE-CF594-A :: RFP
Comp-FITC-A :: GFP
(P2)
(P1)
0.95
9.61
8.60
LdWT
LdCen-/-
(P1)
(P2)
(P1)
(P2)
Count
48.3
17.0
64.2
18.1
CD40
B
P2-DC (CD11c+ MHCIIhiGFP-RFP+) + CD4 T cell
UI
LdWT
LdCen-/-
Modal
1.6547
1.6
2.0353
2.0
2.7518
2.7

***Supporting Information S3: Negligible T cell proliferation was observed in infected P2-DC and CD4T cell coculture set. (A)*** *Gating strategy for flow cytometry analysis and representative flow plots showing the expression of CD40 in P1 and P2 DCs in ear dLN of LYS-eGFP infected mice.* ***(B)*** *Ag-specific CD4 T cell proliferation was estimated from P2 DC–CD4 T cell coculture assay by studying CFSE dilution of gated $CD4^{+}CD44^{+}$ T cells and is represented by the histogram.*
