# Supplementary material for: Neutrophil-dendritic cell interaction plays an important role in live attenuated Leishmania vaccine induced immunity
Source: PLoS Negl Trop Dis. 2022 Feb 22;16(2):e0010224. doi: 10.1371/journal.pntd.0010224 (PMC8896671; doi:10.1371/journal.pntd.0010224)
Supplement: S4 Fig — (A) The gating strategy and individual flow plots for DCs (Cd11b+Cd11c+Ly6G-Ly6C-MHCIIhi) recruitment in ear dLN has shown at 5-day post infection in mice depleted or not of neutrophils. (PDF) [file pntd.0010224.s004.pdf]

***S*4 Fig**

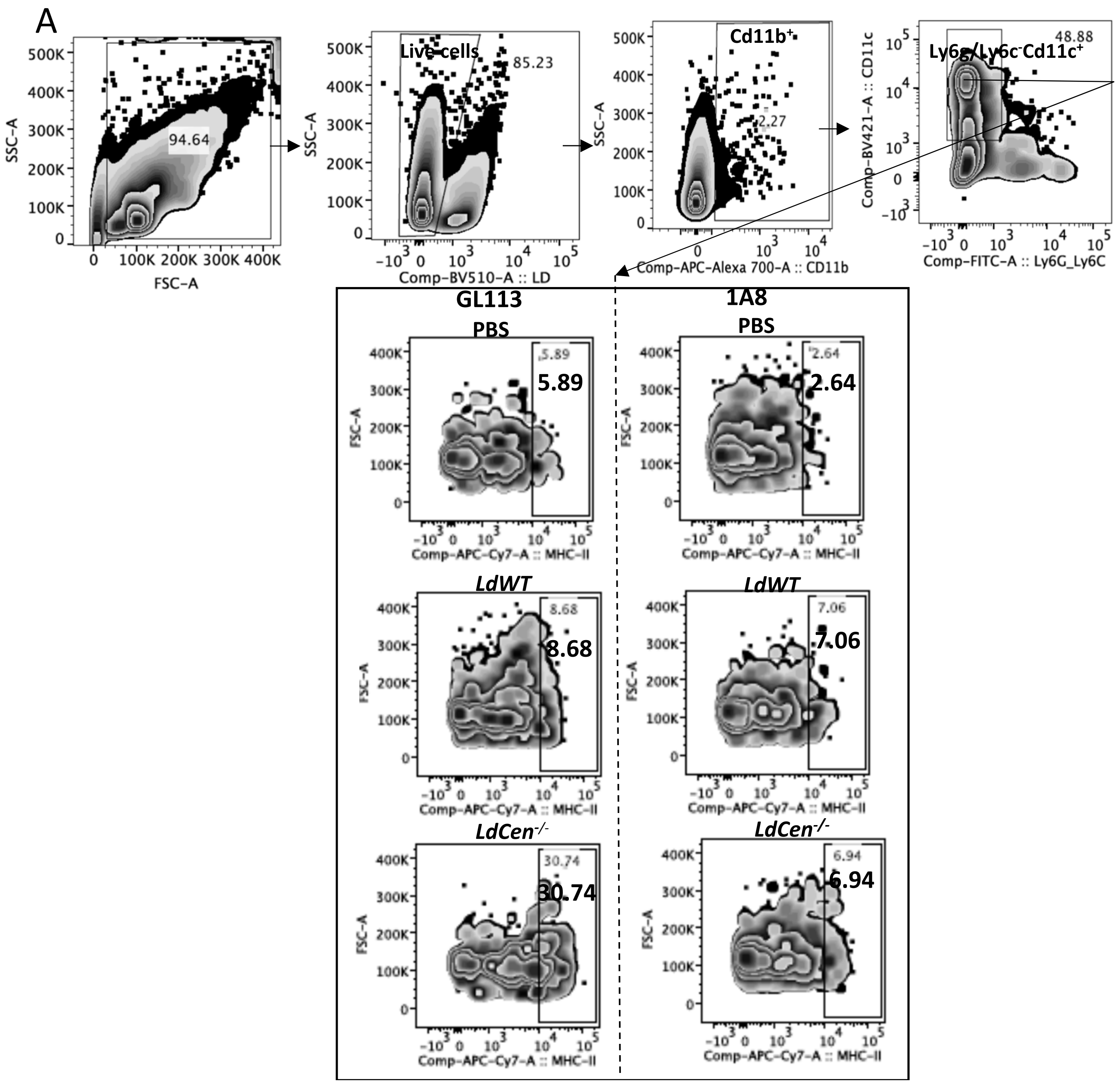
A
94.64
SSC-A
FSC-A
Live cells
85.23
Comp-BV510-A :: LD
Cd11b+
2.27
Comp-APC-Alexa 700-A :: CD11b
Ly6g/Ly6c-Cd11c+
48.88
Comp-BV421-A :: CD11c
Comp-FITC-A :: Ly6G_Ly6C
GL113
1A8
PBS
5.89
2.64
LdWT
8.68
7.06
LdCen-/-
30.74
6.94
FSC-A
Comp-APC-Cy7-A :: MHC-II

Supporting Information S4: Kinetics of DC recruitment in GL113 or 1A8 treated mice following infection with LdWT/LdCen$^{-/-}$ *(**A**) The gating strategy and individual flow plots for DCs (Cd11b^{+}Cd11c^{+}*Ly6G^{-}Ly6C^{-}*MHCII^{hi}) recruitment in ear dLN has shown at 5-day post infection in mice depleted or not of neutrophils.*
