# Supplementary material for: Neutrophil-dendritic cell interaction plays an important role in live attenuated Leishmania vaccine induced immunity
Source: PLoS Negl Trop Dis. 2022 Feb 22;16(2):e0010224. doi: 10.1371/journal.pntd.0010224 (PMC8896671; doi:10.1371/journal.pntd.0010224)
Supplement: S5 Fig — (A) Total number of Cd11b+ cells recruited in ear dLN 5 days post infection in either GL113 or 1A8 treated mice has been shown. The experiment was repeated three times with pooled digests from five to six ear dLNs per experiment. The data represent the mean values ± SD of results from three independent experiments that all yielded similar results. (B, C) The percentages of CD80 and CD40 positive DCs in ear dLN of GL113/1A8 treated LdWT/LdCen-/- infected mice 5d post infection was reported. (D) Gating strategy showing CFSE dilution of gated CD4+CD44+T cells from DC-CD4 T cell coculture assay. (PDF) [file pntd.0010224.s005.pdf]

S5 Fig

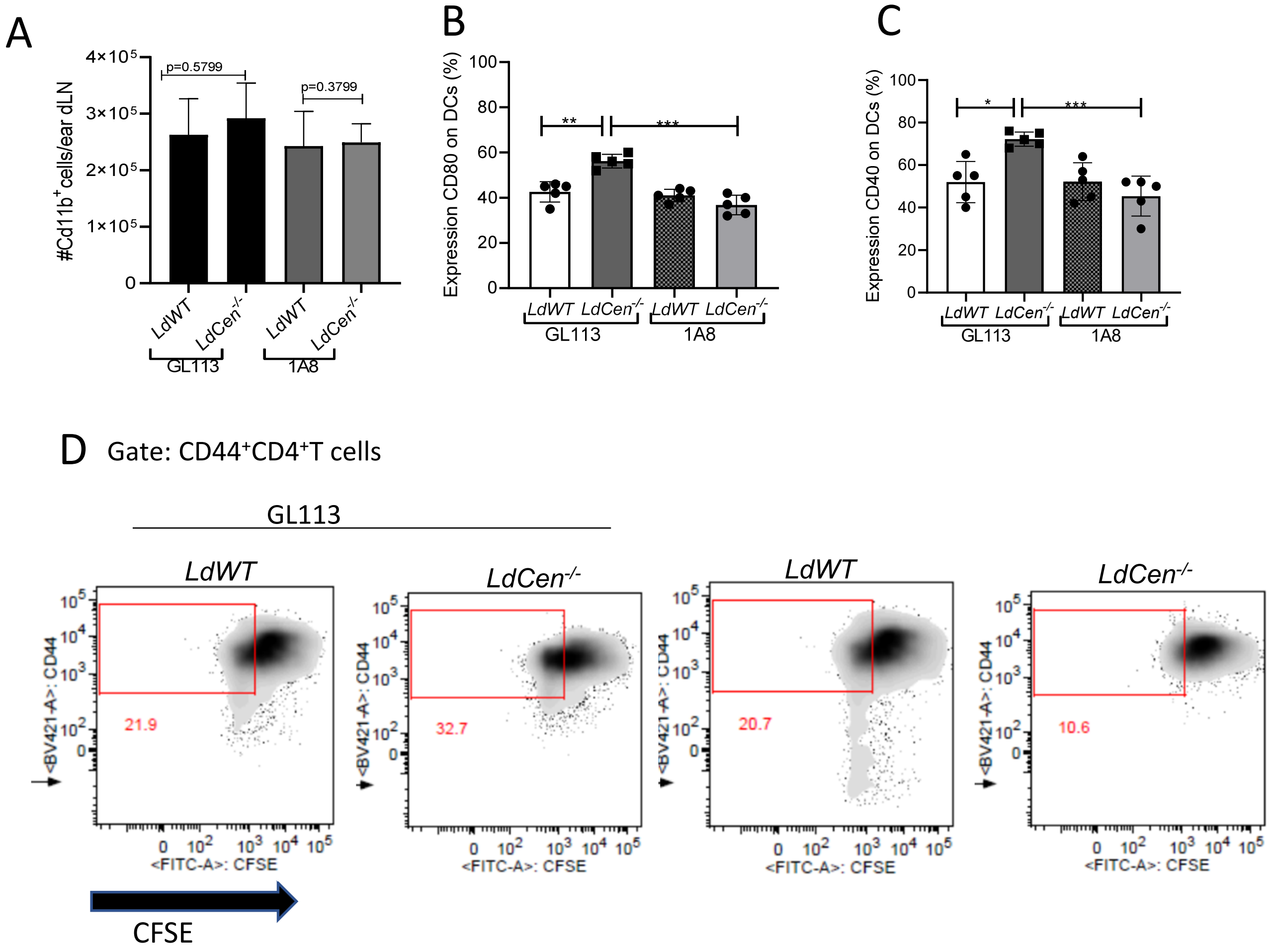
A
#Cd11b⁺cells/ear dLN
p=0.5799
p=0.3799
LdWT
LdCen-/-
GL113
1A8
B
Expression CD80 on DCs (%)
**
***
C
Expression CD40 on DCs (%)
*
***
D Gate: CD44⁺CD4⁺T cells
GL113
LdWT
LdCen-/-
21.9
32.7
20.7
10.6
<BV421-A>: CD44
<FITC-A>: CFSE
CFSE

Supporting Information S5: DCs from neutrophil depleted LdCen$^{-/-}$ infected mice exhibited compromised CD4T cell priming ability ex-vivo. (A) *Total number of Cd11b^{+} cells recruited in ear dLN 5 days post infection in either GL113 or 1A8 treated mice has been shown. The experiment was repeated three times with pooled digests from five to six ear dLNs per experiment. The data represent the mean values ± SD of results from three independent experiments that all yielded similar results.* ***(B,C)*** *The percentages of CD80 and CD40 positive DCs in ear dLN of GL113/1A8 treated LdWT/LdCen$^{-/-}$ infected mice 5d post infection was reported.* ***(D)*** *Gating strategy showing CFSE dilution of gated CD4^{+}CD44^{+}T cells from DC-CD4 T cell coculture assay.*

.
