# Supplementary material for: Neutrophil-dendritic cell interaction plays an important role in live attenuated Leishmania vaccine induced immunity
Source: PLoS Negl Trop Dis. 2022 Feb 22;16(2):e0010224. doi: 10.1371/journal.pntd.0010224 (PMC8896671; doi:10.1371/journal.pntd.0010224)
Supplement: S6 Fig — (A) Schematic diagram showing experimental scheme for neutrophil depletion and subsequent experiments. (B) Real-time PCR analysis of RNA isolated from purified CD4 LN T lymphocytes at 5d post infection as described in Materials and Methods is shown. Normalized expression levels of Tbet were estimated. Data are presented as fold change from uninfected naive mice. The data represent the mean values ± standard deviations of results from 3 independent experiments that all yielded similar results. *, P < 0.05; **, P < 0.005. (n = 6). (C) Parasite numbers in ear dLNs of different groups of GL113/1A8 treated infected mice were measured 7 days post infection. Means and standard errors of the means for 5 mice in each group are shown. Data are representative of two independent experiments. *, P < 0.05. (PDF) [file pntd.0010224.s006.pdf]

*S*6 Fig

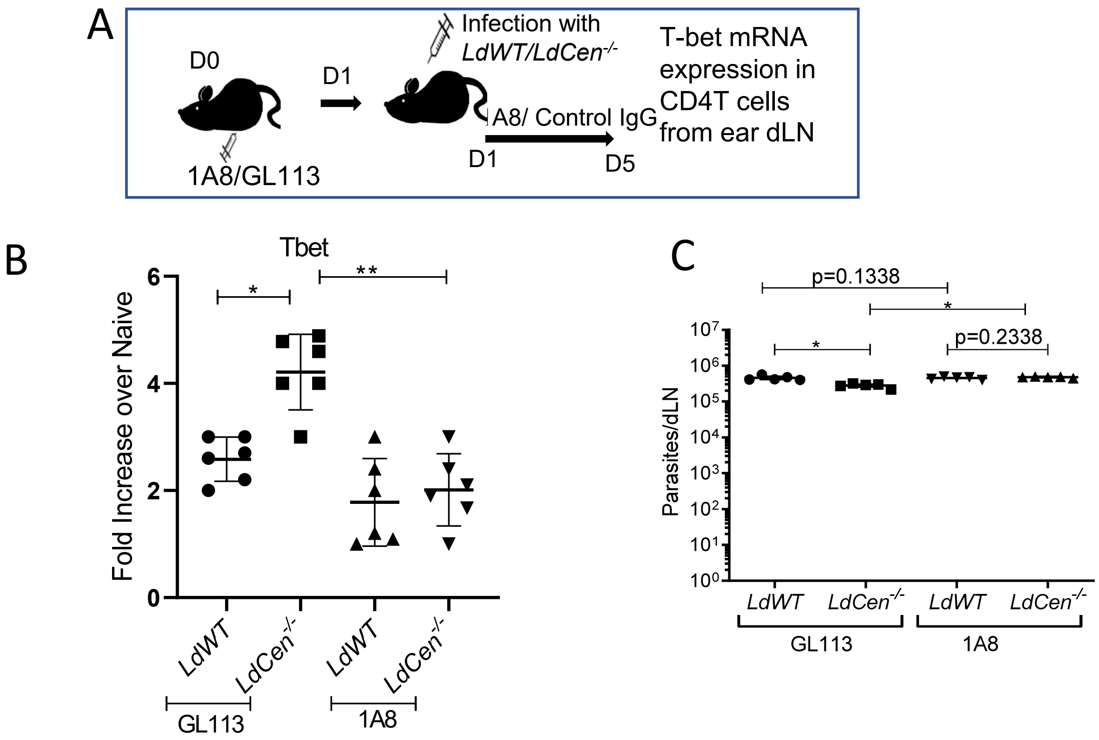
A
D0
1A8/GL113
D1
Infection with
LdWT/LdCen-/-
A8/ Control IgG
D1
D5
T-bet mRNA expression in CD4T cells from ear dLN
B
Tbet
Fold Increase over Naive
*
**
0
2
4
6
LdWT
LdCen-/-
LdWT
LdCen-/-
GL113
1A8
C
p=0.1338
*
*
p=0.2338
Parasites/dLN
LdWT
LdCen-/-
LdWT
LdCen-/-
GL113
1A8

Supporting Information S6:

Neutrophil depleted immunized mice ear dLN derived CD4 T cells exhibits attenuated Tbet expression and exacerbated parasite numbers

(A) *Schematic diagram showing experimental scheme for neutrophil depletion and subsequent experiments. (**B**) Real-time PCR analysis of RNA isolated from* purified *CD4 LN T lymphocytes at 5d post infection as described in Materials and Methods* is shown. *Normalized expression levels of Tbet was estimated. Data are presented as fold change from uninfected naive mice. The data represent the mean values ± standard deviations of results from 3 independent experiments that all yielded similar results.*, $P < 0.05$; **, $P < 0.005$. (n=6).* ***(C)*** Parasite numbers in ear dLNs of different groups of GL113/1A8 treated infected mice were measured 7 days post infection. Means and standard errors of the means for 5 mice in each group are shown. Data are representative of two independent experiments. **, $P < 0.05$.*
